# Can we ever have evidence-based decision making in orthopaedics? A qualitative evidence synthesis and conceptual framework

**DOI:** 10.1186/s12911-025-03032-5

**Published:** 2025-07-01

**Authors:** Arabella Scantlebury, Katherine Jones, Joy Adamson, Melissa Harden, Catriona McDaid, Amy Grove

**Affiliations:** 1 Centre for Evidence and Implementation Science, College of Social Sciences, University of Birmingham, Birmingham, B15 2RT England; 2 Warwick Medical School, University of Warwick, Warwick, CV4 7AL England; 3 York Trials Unit, Department of Health Sciences, University of York, York, YO10 5DD England; 4 Centre for Reviews and Dissemination, University of York, York, YO10 5DD England

**Keywords:** Surgery, Evidence-based practice, Implementation, Decision-making, Orthopaedics, Evidence-based medicine, Qualitative evidence-synthesis, Qualitative

## Abstract

**Background:**

The perception and use of scientific evidence in orthopaedic surgical decision-making is variable, and there is considerable variation in practice. A previous conceptual framework described eight different drivers of orthopaedic surgical decision-making: formal codified and managerial knowledge, medical socialisation, cultural, normative and political influence, training and formal education, experiential factors, and individual patient and surgeon factors. This Qualitative Evidence Synthesis (QES) aims to refine the conceptual framework to understand *how* these drivers of decision-making are applied to orthopaedic surgical work in a dynamic and fluid way.

**Methods:**

A QES explored how different types of knowledge and evidence inform decision-making to explore why there is so much variation in orthopaedic surgical work. Nine databases were systematically searched from 2014 to 2023. Screening was undertaken independently by two researchers. Data extraction and quality assessment were undertaken by one researcher and accuracy checked by another. Findings were mapped to the conceptual framework and expanded through thematic synthesis.

**Results:**

Twenty-five studies were included. Our re-conceptualised framework of evidence-based orthopaedics portrays *how* surgeons undergo a constant process of medical brokering to make decisions. Routinely standardising, implementing and regulating surgical decision making presents a challenge when the decision-making process is in a constant state of flux. We found that surgeons constantly prioritise drivers of decision-making in a flexible and context-specific manner. We introduce the concept of socialisation in decision making, which describes “the socialisation of factors affecting decision-making. Socialisation is additive to surgeon identity and organisational capacity, which as explanatory linchpins act to mediate our understanding of how and why surgical decision-making varies. Our conceptual framework allows us to rationalise why formal codified knowledge, typically endorsed through clinical guidelines, consistently plays a limited role in orthopaedic decision-making.

**Conclusions:**

We present a re-conceptualised framework for understanding what drives real world decision-making in orthopaedics. This framework highlights the dynamic and fluid way these drivers of decision-making are applied in orthopaedic surgical work. A shift in orthopaedics is required away from prioritising informal, experiential knowledge first to incorporating evidence-based sources of evidence as essential for decision-making. This paradigm shift, views decision-making as a complex intervention, that requires alternative approaches underpinned by multi-faceted, evidence-based implementation strategies to encourage evidence-based practice.

**Registration:**

PROSPERO CRD42022311442

**Clinical Trial Number:**

Not applicable.

**Supplementary Information:**

The online version contains supplementary material available at 10.1186/s12911-025-03032-5.

## Background

Evidence-based practice (EBP) implies that clinical expertise; the proficiency and judgement that clinicians acquire through their experience, is combined with the best available external clinical evidence to inform practice. Thirty years on from the inception of EBP, there remains considerable variation in surgical practice and differential use of clinical evidence in decision-making [1–3].

Orthopaedic surgery is a specialty renowned for resisting standardisation [2, 4–7]. Historically, the surgical community has attributed the limited role of clinical evidence in their decision-making to a lack of high quality, robust (e.g. Randomised Controlled Trial) evidence and/or lack of equipoise [8, 9]. Orthopaedic surgery is becoming increasingly research active and surgeons and surgical teams are obtaining multi-million pound, publicly funded research investment to support orthopaedic research [10, 11]. There are numerous examples in orthopaedic research, which demonstrate the positive steps being made towards increasing evidence-based practice. Getting it Right First Time (GIRFT) is a national programme funded by NHS England. GIRFT uses national benchmarking datasets to identify and tackle variation to drive practice change [12]. Internationally, there are distinct areas of orthopaedic research which are creating substantial evidence to practice change, for example; antibiotic prophylaxis and treatment of fractures to the scaphoid waist, proximal humerus and primary frozen shoulder [13–16]. Unfortunately, history shows us that the production of high quality research evidence and guideline recommendations are not enough to change practice [3, 6]. To demonstrate a return on this growing research investment, avoid research waste and reduce clinical uncertainty, we need to understand why the perception and application of evidence in orthopaedics remain uncertain and variable [3, 4, 9].

A mixed methods systematic review explored what factors influence decision-making and practice variation in orthopaedic surgery [4]. Findings were presented in a conceptual framework which highlighted the limited role of formal codified knowledge (e.g. clinical evidence and guidelines) in the practice of surgeons. The predominantly quantitative review identified a complex set of factors which compete to influence decision-making. We refer to these factors as the ‘what’ and they included: formal codified and managerial knowledge, medical socialisation, cultural, normative and political influence, training and formal education, experiential factors, individual patient and surgeon factors.

Since the original review was published in 2014, where only four of 24 included studies were qualitative in nature, there has been a sharp increase in qualitative orthopaedic research. (see Appendix 1) [9, 17, 18] This more exploratory research has grown in parallel to the rise in demand for orthopaedic services. Whilst COVID-19 pandemic elective backlogs contribute to service demand, a more fundamental issue is the mismatch between the requirements of an ageing population and orthopaedic service capacity. The urgency to ensure clinical and cost-effective surgical interventions foregrounds our study, which aims to refine an orthopaedic decision-making conceptual framework to understand *how* these factors are applied to orthopaedic surgery in practice.

We undertook a Qualitative Evidence Synthesis (QES) to update and expand the original review to crystalise our understanding of *how* different evidence and knowledge sources compete and are used in surgical decision-making. We set out to explore *why* there is such a limited role for research evidence implementation in surgical practice, despite the financial investment and upward trend in the conduct of orthopaedic research in high income countries.

## Methods

The review was prospectively registered on PROSPERO (CRD42022311442) and conducted and reported in accordance with the Cochrane Qualitative and Implementation Methods Group guidance for conducting QES [19] and the ENTREQ (Enhancing Transparency in Reporting the synthesis of qualitative research) statement [20]. The PRISMA 2020 Checklist is provided in additional file [2].

### Search strategy

Our information specialist (MH) updated the original search strategy [4] and incorporated search terms for additional eligibility criteria. The MEDLINE strategy was tested and refined to capture both the original included studies and recent key studies we were aware of. It was peer reviewed by a second information specialist using the Peer Review of Electronic Search Strategies (PRESS) checklist [21] and translated for other databases. (Additional file 3) Retrieval was restricted to English language studies. A date limit of 2014 was applied to identify studies post-2014 which was the search end date for the original review.

Nine databases were searched by an information specialist on 19th January 2022 (updated 21st March 2023): MEDLINE ALL (Ovid), Embase (Ovid), PsycINFO (Ovid), CINAHL Plus (Ebsco), ASSIA (Proquest), Science Citation Index (Web of Science), Social Science Citation Index (Web of Science), Cochrane Central Register of Controlled Trials (Wiley) and the Cochrane Database of Systematic Reviews (Wiley).

### Eligibility criteria

We adopted the PeRSPE(C)TiF tool to describe our eligibility criteria (Table 1) [22]. We aimed to identify the type of evidence and knowledge that orthopaedic surgeons and/or surgical staff use to inform their decision-making. This included but was not limited to: patient characteristics (age, lifestyle, pain), formal evidence sources (scientific evidence) and surgeon characteristics (surgeon experience, specialty norms).

**Table 1:** QES eligibility criteria as described using the PerSPE(C)TiF tool [22]

PerSPE(C)TiF Term	Qualitative Evidence Synthesis Definition
Perspective	Orthopaedic surgeons and orthopaedic surgical team (surgical staff, nurses, allied health professionals) members involved in surgical decision-making.
Setting	Any orthopaedic surgical procedure conducted in adult and paediatric services in any hospital setting, academic, community and public services. No country restriction was applied.
Phenomenon/Problem	What factors influence decisions in orthopaedic surgical work.
Environment (Optional Comparison)	Any service where orthopaedic surgical decision making occurs (No comparator)
Time/timing	The time period when orthopaedic surgical decisions are made (pre-surgery). Decision making surrounding referral for initial conservative management prior to surgery is not included. For example, referral to lifestyle programme management by Primary Care.
Findings	With relevance to researchers, policy makers, clinicians and patients.

Studies were included if they used established methods of qualitative data collection (e.g., interviews, focus groups) and analysis (e.g., thematic analysis). Mixed methods studies were included if they had an identifiable qualitative component suitable for extraction. Studies with mixed populations, i.e., multiple surgical specialties were included provided it was possible to separately extract the results for our population of interest.

### Study selection and data extraction

Records were downloaded into COVIDENCE [23]. Two researchers (KJ, AS) independently screened titles and abstracts and full papers, recording reasons for exclusion. Discrepancies were resolved by consensus or discussion with a third researcher (JA).

Two data extraction forms were created within Microsoft Excel [24]: (1) study characteristics and (2) findings (Table 2). Two researchers independently extracted two records to ensure consistency (KJ, AS), remaining articles were extracted by one researcher (KJ) and accuracy checked by a second (AS).

**Table 2:** Details of data extraction

Study characteristics	Country, author, ‘intervention’ delivered (surgery type and condition), setting and staff characteristics (age, gender, ethnicity, specialty, years experience and grade)
Study details	Aims and objectives, design, recruitment and sampling strategies, sample size and methods of data collection and analysis
Author interpretations	Any information (participant quotations and author interpretations) reported in the results sections of included studies that described how surgical decisions are made in orthopaedic surgery

### Quality assessment

The Critical Appraisal Skills Programme (CASP) checklist [25] was used for quality assessment as recommended by Cochrane Qualitative Implementation Methods group guidance [19]. Each article was assessed by one reviewer (KJ) and checked by a second (AS). Discrepancies were resolved through discussion.

### Data synthesis

We undertook a manual thematic synthesis which iteratively moved between coding and theme development [26]. Initially, we produced thematic tables and performed deductive coding, broadly, and descriptively coding data according to the eight types of knowledge and evidence identified by the original review [4]. Separate word documents were created for each knowledge type (Table 3):

**Table 3:** Example thematic table

Formal codified knowledge			
Reference	Theoretical category as described in the original review (e.g. National guidelines, academic journal articles)	Author interpretation	Quotation

Thematic tables were then analysed similarly to interview transcripts within primary qualitative studies and provided a basis for more in-depth thematic analysis of each knowledge type. This involved constant comparison between our findings and that of the original review and we revisited studies included in the original review [4]. A key analytic challenge was conceptualising the interrelated nature of data, particularly for knowledge types and data that went beyond the original conceptual framework (e.g., surgeon identity and organisation). We compared and contrasted data across and within thematic tables, to explore how identity and organisation could explain how different knowledge sources compete and are used in decision-making.

## Results

### Characteristics of included studies

**Fig. 1:**
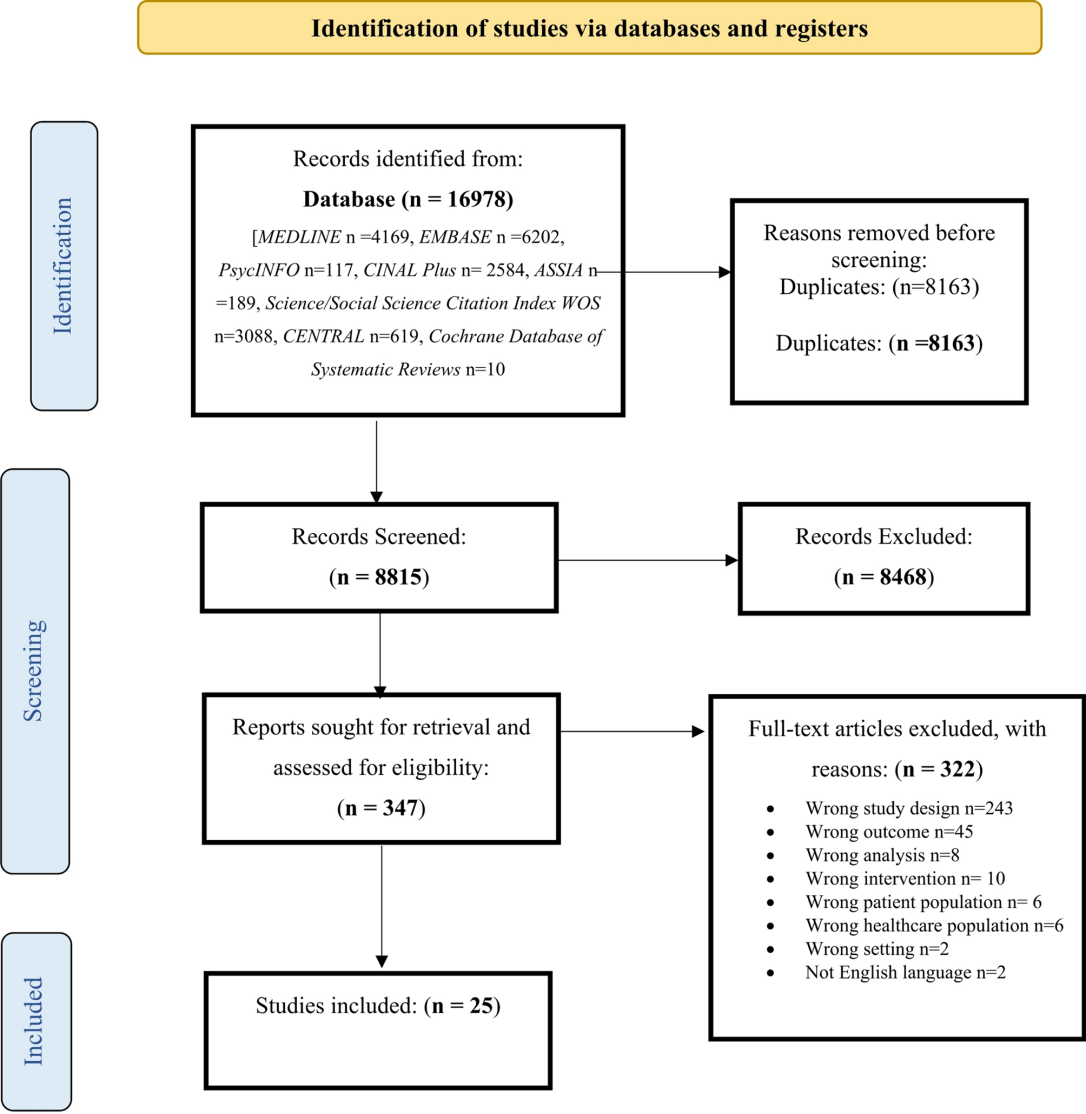
PRISMA flow diagram of literature search and study selection phases

A total of 8,815 records were screened for inclusion in the QES (Fig. 1).

25 studies were included: 21 qualitative studies and four mixed methods studies (see Table 4 for contextual information and additional file 1 for full study characteristics).

**Table 4:** Summary of key contextual information from included studies

Reference (year) Country	Country (Setting*)	Participant characteristics* (Total *n*)	Orthopaedic procedure(s)
Adogwa et al., (2021)	U.S (Hospital, single site)	(*n* = 11 *n* = 6 patients, *n* = 5 spine surgeons) Years of experience: average years in practice 5.	Surgery for correction of adult spinal deformity
Baker et al. (2019)	US (Hospital multi-site)	(*n* = 2, 9 orthopaedic surgeons, 3 hospitalists, 3 geriatricians, 5 nurses, 3 occupational therapists, 3 physical therapists and 2 clinical ethicists.) Gender: 14 Females, 11 Males, 3 unspecified.	Surgical interventions for patients with hip fractures who are hospitalised with dementia
Barton et al. (2021)	US (“Physicians from across North America”)	(*n* = 14 orthopaedic surgery *n* = 6; neurosurgery *n* = 5, radiation oncology *n* = 2 and physiatry *n* = 1) Training level (grade): All faculty levels from instructor to professor. Gender: 11 Male, 3 Female	Operative and non-operative management for spinal metastatic disease
Brown et al. (2018)	South Africa (Public tertiary teaching hospital)	(*n* = 23 orthopaedic consultants and registrars 9; registered nurses from orthopaedic wards, clinics and pain service 5, allied health professionals (physiotherapists, occupational therapists, dieticians, social worker) 9). Ethnicity: White 13, Indian 4, Africal (isiZulu) 4, Mixed race 2. Gender: 15 Females, 8 Males.	Treatment of osteosarcoma
Bunzil et al. (2017)	Australia (Single, tertiary teaching hospital)	(*n* = 20 orthopaedic surgeons) Training level (grade): 15 consultants, 5 registrars. Years experience: of performing Total Knee Arthroplasty (TKA) ranged from 6 months to 30 years.	Surgery for total knee arthroplasty
Bunzil et al. (2021)	Public and private hospitals in 14 countries.	(*n* = 18 specialist sarcoma surgeons)Years experience: average 19 years as a sarcoma surgeon.	Orthopaedic oncology surgeries
Coole et al. (2021)	England (NHS Hospital multi-site)	(*n* = 40 12 allied health professionals, nurses 95 Occupational Therapists, 4 Physiotherapists and 3 nurses), 12 orthopaedic surgeons and 16 General Practitioners). Training level (grade): surgeons: 12 consultants, GPs: 14 partners, 1 salaried, 1 registrar, AHPs and Nurses: Band 7, 8, Band 6, 3, Band 5, 1. Years experience: 1–32 years. Male: 26, Female: 14	Treatment of total knee replacement and total hip replacement
Dismore et al. (2021)	UK (NHS hospital single site)	(*n* = 14 orthopaedic practitioners) Training level (grade): 11 consultants, 3 registrars. Years experience: 3–25 years of orthopaedics Age: range 29–52 years. Female: 2, Male 12	Forefoot surgery for hallux valgus (bunion) or hallux rigidus (arthritis of the big toe joint)
Frankel et al. (2016)	Canada (Community hospital and academic centre – academic and urban centres)	(*n* = 14 orthopaedic surgeons) Male 12, Female 2. Age: <50 7; 51–64 6, 65 + 1	Total joint arthroplasty
Grove et al. (2018)	UK (3 English NHS hospitals)	(*n* = 121) (clinical 34, allied health professionals, 17 managers and national stakeholders 13) (*n* = 64) supplementary documents	Total hip replacement for end-stage arthritis
Grove et al. (2020)	England (Three NHS hospitals in England.)	(*n* = 121) (clinical 34, allied health professionals, 17 managers and national stakeholders 13) (*n* = 64) supplementary documents	Orthopaedic surgery.
Grove et al. (2021)	England (Three NHS hospitals in England.)	(*n* = 121) (clinical 34, allied health professionals, 17 managers and national stakeholders 13) (*n* = 64) supplementary documents	Hip arthroplasty
Haider et al. (2020)	England (Hospital based trauma and orthopaedic departments)	(*n* = 113 regional trainee representatives of the British Orthopaedic Trainees Association)	Trauma meetings in orthopaedic surgery
Hsu et al. (2017)	USA (“Orthopaedic providers”)	(*n* = 19 orthopaedic and cardiology clinicians) M: 15 F: 4 (Year 1) M: 11, F: 4 (Year 2)	Implementation of decision aid in cardiology and orthopaedics
Jefferson et al. (2017)	UK (NHS, major trauma centre or equivalent tertiary hospital, trauma unit, or secondary care hospital, district hospital or other)	(*n* = 265 surgeon members of the British orthopaedic association and British elbow and shoulder society) Training level (grade): consultant: 218; speciality trainee ST7 or ST8 18, Speciality trainee ST1 or ST6 11, staff associate specialists 10, fellows 6, missing 1. Years experience of treating fractures of the proximal humerus: 0–5 years 25; 6–10 years 82; 11–15 years; 57; 16–20 years 41 > 21 60. Male 249, Female 16. Age: <35: 23, 36–45 98, 46–55 88, 56–65 54 > 66 2.	Surgical and non-surgical treatment for adults with displaced proximal humerus fractures involving the surgical neck
Madsen et al. (2021)	Denmark (Orthopaedic outpatient shoulder clinic, Silkeborg regional hospital)	(*n* = 7 3 extended scope physiotherapists and 4 orthopaedic surgeons) Training level (grade): consultant 3 and registrar 1. Physiotherapist grade not reported. Years experience: all had more than 3 years of experience at the shoulder clinic, Silkeborg hospital.	Diagnosis and treatment for patients with shoulder disorders
Moore et al. (2017)	England and Wales (5 high volume NHS orthopaedic departments)	(*n* = 12 male orthopaedic surgeons) Training level (grade): all consultants Years experience: average 14 years treating prosthetic joint infection. Age: Average 49 years.	Revision surgery for Prosthetic joint infection after hip arthroplasty.
Phelps et al. (2019)	England (7 NHS hospitals)	(*n* = 24 staff were interviewed, ten surgeons and 14 research associates which included nurses an RA a physiotherapist a research manager and trial coordinator).	Intradeullary nails versus distal locking plates for fractures of the distal femur
Rath et al. (2017)	India (major public tertiary care hospitals)	(*n* = 11 with key informants, clinical leads, residents and nursing staff from orthopaedics, anaesthesia, geriatrics, medicine and physiotherapy).	Hip fractures in older adults
Rehman et al. (2019)	Canada (Neurosurgical practices *n* = 6 Ontario)	*n* = 18 (12 patients, 6 neurosurgeons) Years experience: range 8–26 Age: Range 45–68 years	Risks and benefits of lumbar decompressive surgery (LDS) for sciatica
Robba et al. (2019)	UK (Consultant hand surgeons UK-wide)	*n* = 10 Consultant hand surgeons	Treatment of triangular fibrocartilage complex (TFCC)injuries
Scantlebury et al. (2022)	UK hospitals (*n* = 13)	*N* = 19 spinal surgeons. Male: 14 Female: 5	Stable thoracolumbar fractures without spinal cord injury
Schmidtke et al. (2022)	Acute hospitals in England	*N* = 25 academics: 6, surgeons: 9, other 23 (dietician, speech and language therapists, radiologist, gastroenterologist, general practitioner) involved in six surgical trials in stroke, gastro-oesophageal reflux disease, abdominal aortic aneurysm, knee replacement, varicose veins (2 trials). Years experience: median 20	KAT trial – patella resurfacing
Shaw et al. (2022)	UK hospitals undertaking major orthopaedic, colorectal and/or cardiac surgery	(*n* = 31 patients, 19 relatives and 37 surgeons and anaesthetists (21) representing orthopaedics (*n* = 3), colorectal (8) and/or cardiac (3) surgery)	High risk patients offered major surgery. Orthopaedic patients (hip and knee)
Sutton et al. (2021)	Australia (Urban and rural GP and orthopaedic practices Tasmania)	(*n* = 27) General Practitioners (17) and orthopaedic surgeons (10) Age: range 33–62 Gender: 4 GPs and all surgeons were male.	Conservative and surgical management for osteoarthritis

*As described by study authors

Included studies were undertaken in the UK (*n* = 13), USA (*n* = 4), Canada (*n* = 2), Australia (*n* = 2), South Africa (*n* = 1), Denmark (*n* = 1), and India (*n* = 1). One study reported findings from across 14 countries [27]. Studies discussed surgical decision-making across a range of orthopaedic conditions, most commonly hip and knee fractures and/or replacements (*n* = 10). 19 of our included studies stated that qualitative researchers had been involved in data collection and/or analysis. The backgrounds of the authors in the remaining 6 studies were unclear and inconsistently reported. Orthopaedic specialties represented within primary studies included: hip and knee fractures and/or replacements (*n* = 10), spinal surgery (*n* = 4); orthopaedic oncology (*n* = 2), osteoarthritis (*n* = 1), foot and ankle surgery (*n* = 1), elbow and shoulder surgery (*n* = 1), shoulder disorders (*n* = 1), upper leg (= 1) hand and wrist (*n* = 1) and mixed multiple specialties across orthopaedics (*n* = 3) Study participants included surgeons (orthopaedic and spinal), anaesthetists, allied health professionals (physiotherapists, occupational therapists, clinical ethicists, managers), nurses and general practitioners. The views of surgeons dominated the data, and most participants were male. Gender, age, ethnicity, years’ experience and healthcare funding model were poorly and inconsistently reported.

Three of the included studies used data from the same qualitative dataset and are treated as separate papers as they report on different aspects of surgical decision making [5, 17, 28]. Most studies were interview studies (*n* = 23) and/or analysed data thematically (*n* = 19).

### Quality appraisal

We supplemented the CASP tool [25] (additional file 4) with a narrative description of study quality. This helped us to consider recent qualitative methodological advances and provide more detailed reflection of the methodological issues in our included studies.

Study quality varied hugely. Of those that discussed sampling, many used opportunistic approaches such as snowballing. We consider this preference for snowballing appropriate and pragmatic for orthopaedic surgical research, where the potential sample is small, and surgeons in the community are known to each other.

Historically, journal word limits have prohibited detailed descriptions of qualitative analysis - this is a particular problem for medical journals, where 19 (90%) of our included studies were published. We identified a reliance on using the phrase “*thematic analysis*” and/or citation of Braun and Clarke’s method as a way of describing the analysis process [29]. Other analysis descriptions were insufficient, and often did not cite an analytical method for example “*inductive with themes derived from the data”*. In these circumstances, we made subjective judgements about use of an *established qualitative method of analysis (see eligibility criteria section)* according to the data presented in the methods and results sections.

### Thematic synthesis findings

**Fig. 2:**
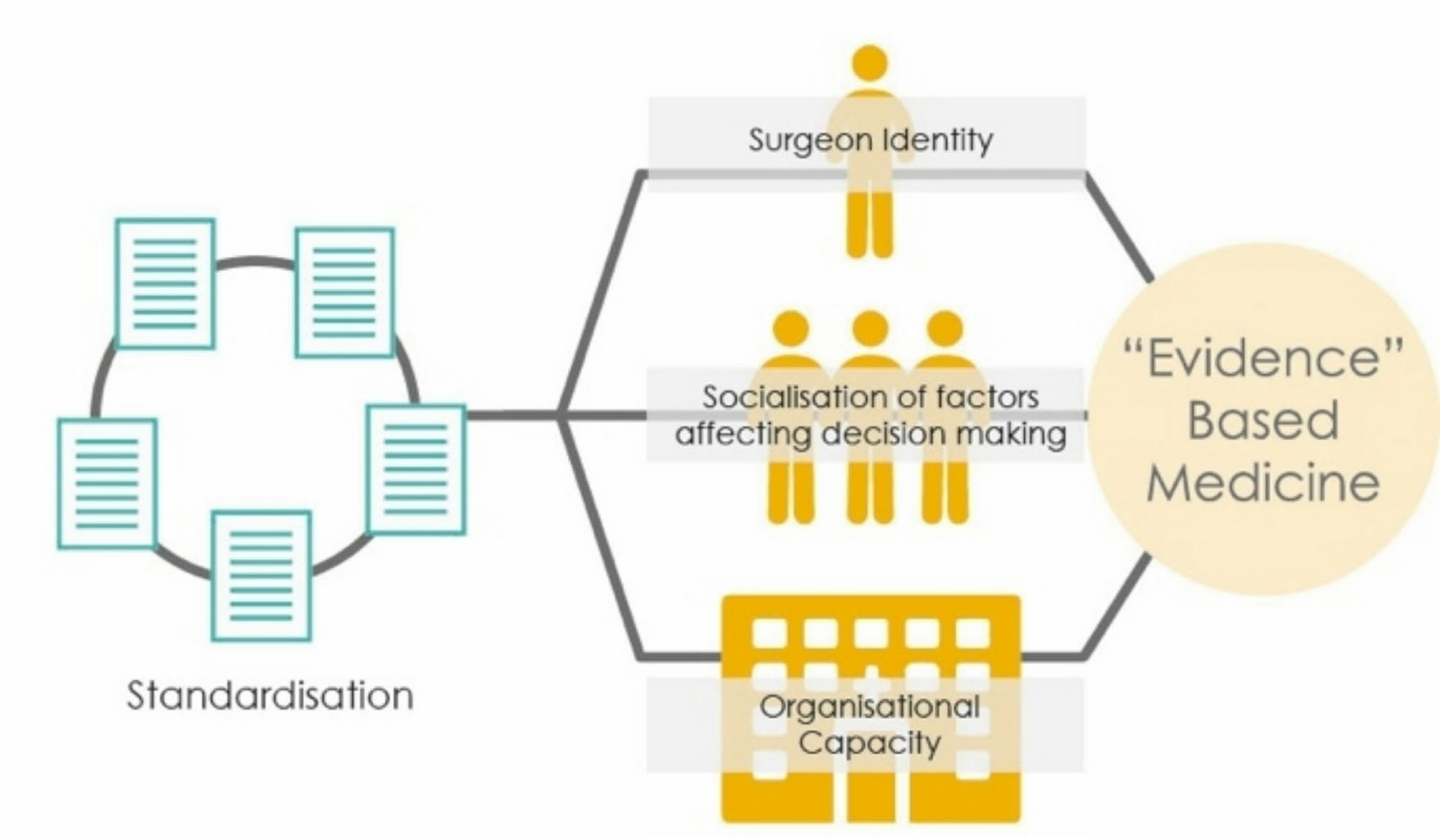
A conceptual framework of evidence-based orthopaedics: the linchpin model

Our over-arching finding is that practice variation and the limited role of formal codified knowledge in decision-making is central to understanding the stymied progression towards standardisation in orthopaedics. First, we explore the role of formal codified knowledge in decision-making, before expanding on the remaining sources of evidence and knowledge that appear to influence orthopaedic decision-making (exemplar quotes are provided in additional file 5). Whilst we report our findings in a static, two-dimensional, sequential format, the factors which influence decision making constantly overlap, compete, change and are socialised in context. (see Fig. 2) This consistent brokering of knowledge around a decision implies that our findings could be placed under multiple theoretical constructs and our framework should be interpreted flexibly. A challenge with flexible interpretation is simultaneously conveying *what* evidence and knowledge influence decision-making, whilst illuminating *why* evidence sources vary so substantially for each patient and/or surgical decision.

Our “*Conceptual Framework of Evidence-Based Orthopaedics: The Linchpin Model*” (Fig. 2), portrays decision-making as a process that involves surgeons engaging in a constant medical brokering. Decisions are made within a competing set of inter-woven, context-dependent drivers of decision-making situated in a complex evidence economy [30, 31]. This “socialisation of factors affecting decision-making” represents the process of progressing each knowledge and evidence source over one another for surgical decisions. Our re-conceptualisation of decision-making deliberately avoids citing each individual driver of decision-making (see appendix 6) to avoid a linear and/or hierarchical depiction of how surgical decisions are made. We found that the component parts of decision-making are not mutually exclusive and should not be represented as such. Our framework introduces two contextual boundaries of decision-making (i) surgeon identity and (ii) organisational capacity (i.e. the resources and culture within a surgeon’s home organisation) which are fundamental to understanding how decision making is enacted in practice. Surgeon identity and organisational capacity are therefore the mediating factors for understanding decision-making, without which everything else “falls apart.” [32] We frame these contextual boundaries as explanatory *linchpins* for the decision-making process and they are discussed throughout our findings to emphasise how each type of evidence identified in our QES plays out in practice.

### Formal codified knowledge – macro level clinical guidelines and scientific literature

The role of formal codified knowledge in surgical decision-making was emphasised in only three of the 25 studies [3, 33, 34]. Only one study described surgeons as relying heavily on guidelines to inform treatment decisions [33]. We explain this anomaly because the orthopaedic surgeons were part of a wider multi-disciplinary team, treating patients with spinal metastases and working within the scope of oncology, a discipline which participants described as more traditionally evidence-based [33].

The surgical profession tended to differentially value clinical evidence as a primary source of knowledge in decision-making [5, 17]. Evidence appeared to be something which was harnessed to different extents depending on the surgeon’s identity. Professional identities explained surgeon’s different responses to standardisation, and were used to predict whether surgeons are likely to adhere and implement evidence, or not [17].

One study, categorised surgeons by their identity type as either: Mavericks, (“*‘Showmen or women’ unbounded confidence in their surgical ability and self-identified rebel”)* Paragons (“*Gold standard surgeon*s” who perform the same types of surgery using established techniques and describe their practice as evidence based) or Innovators (*“visionaries”* with a desire to try new techniques and implants with ambitions to improve orthopaedic practice) [17]. In this study, Innovators were viewed as *“engineers of orthopaedic surgery*,*”* and although often used evidence, legitimised *“testing the boundaries”* of EBP to ensure the specialty evolved. Mavericks, were seen as mistrustful of scientific evidence and *“didn’t need to know if something worked*,* but needed to judge for themselves that it did*” [17].

The organisational capacity of the surgeon’s current institution also influenced the extent to which formal codified knowledge impacted on decision-making [28]. Organisational capability represents the metaphorical understanding of all knowledge and evidence sources that influence decision-making repeatedly described as *“the way we do things around here”* which fundamentally determined how scientific evidence is, or ever could be implemented in routine practice [9, 28]. Different organisation types, and whether they had a culture towards EBP either facilitated surgeon reliance on implicit knowledge by viewing them as unique or promoted EBP by endorsing the importance of formal codified knowledge. Also important was organisational legacy knowledge, this was described as preferential “*knowledge when surgeons have worked somewhere for a long period of time.”* [28].

*Where* patients receive treatment was a key driver to surgical-decision making, with individual hospitals and/or geographical areas branded as either “*pro-surgery*” or “*pro-conservative treatment*” [9]. In one qualitative case study in England, findings suggested that certain surgeon identities were found to cluster (although not exclusively) around organisations with similar views [28]. This clustering appeared to impact on the use and role of evidence in decision-making and was echoed elsewhere in our findings. We found that University teaching hospitals, or academic centres, typically have a culture of standardisation and potentially attract surgeons and surgical trainees with a disposition towards EBP [28].

However, we found evidence to suggest that studies conducted in specialist orthopaedic centres concluded that scientific evidence regulated through clinical guidelines is limited, and at times irrelevant in this setting, due to their complex, specialist work. Surgeons reported relying on the *“innate feel of surgery”* and their own experiential knowledge to inform decision making [28]. The ability to favour tacit knowledge over explicit evidence was something we found closely tied to organisational norms which in turn, legitimised organisational culture.

On a more practical level, resource availability (e.g. operating theatres) affected decision-making through influencing whether surgeons and the organisation, had the ability to recommend and perform surgery – the organisational capability for EBP. Rath and colleagues, describe how overcrowding and bed shortages in tertiary hospitals in Delhi India, create *“bias against admitting sick patients who require prolonged length of stay.”* [34] Surgeons also described the importance of considering the capacity of other specialties (e.g. microbiology) when considering surgical treatment [34, 35]. Environmental factors such as the physical structure and organisational location of staff influenced decision making. Grove and colleagues [17, 28], observed how orthopaedic services split between two separate geographical sites engendered separate and distinct practice norms and decision-making.

The intersection of surgeon identity and organisation is vital for understanding EBP. As is the notion that surgeon identities are not stable – using a previous example, surgeons could be *“mostly innovators with maverick moments.”* [17] The influence of organisation and identity is variable and dependent on what other knowledge sources are used in each situation. For instance, erratic behaviour may be stifled when working in a University teaching hospital, which is dominated by a high proportion of clinical evidence informed surgeons and a culture of EBP [28].

### Informal experiential implicit knowledge – a surgeon’s gut feeling

“*Clinical intuition”* and surgical experience appeared to be one of the most important and valued influences on orthopaedic surgical decision-making. Our findings describe an overarching mindset that “*what we do in orthopaedics” is “highly specialised knowledge”.* [36] Many studies reported surgeons’ ability to *“predict which patients will do well”* and/or patients’ appropriateness for surgery [9, 14, 17, 28, 35–37]. The role of informal experiential knowledge is intertwined with the philosophical underpinnings of surgery. We found numerous descriptions of surgeons viewing surgery as a craft speciality, or an *“act of faith.*” [17, 33, 36] By framing surgery as an art form, the surgical community emphasise, position, and legitimise their reliance on their own knowledge and are able to reject scientific ideals such as standardisation and the implementation of clinical evidence to orthopaedics [17]. A one-size fits all approach to surgical decision making was strongly opposed by surgeons in several studies. In direct contrast to the principles of RCTs where the standardisation (even for pragmatic trials) of interventions is desirable, surgeons described implementing various forms of experiential knowledge to tailor decisions to the needs of individual patients, and individual fractures. One surgeon described this as the “*personality of the fracture*” which had to be catered for [9, 14, 17, 18, 28, 36–38].

Multiple studies described a perceived lack of robust evidence (e.g., from RCTs) in orthopaedics. This was used as ammunition against EBP and a way to validate a reliance on implicit knowledge [9, 18]. We found studies which asked surgeons “*what would be considered good quality evidence?*” and the responses were hugely variable. Paradoxically, surgeons simultaneously critiqued the robustness and applicability of existing RCT evidence whilst describing their confidence and reliance on observations of colleague’s success rates and case reports [3, 9, 14, 18, 38]. This suggests that the implementation problems we observe in practice [39] do not always stem from the quality of the evidence available in orthopaedics, but the interpretation of the surgical community as to what counts as evidence.

We saw this obliquely in a study which explored the practice impact of surgical trial evidence in England [3]. The study showed that despite an increase in patella resurfacing since trial results were published, significant practice variation remained in line with surgeon training and habits. Despite the perceived *“shift in the community”* towards the use of evidence (e.g., regulation, increase in funding) we found that widespread scepticism towards the appropriateness of scientific evidence persists in orthopaedics.

### Individual patient and surgeon characteristics

#### Patient demographics

A wide range of patient factors (e.g., patient demographics, bone characteristics) were cited when describing patient suitability for surgery (additional file 6). Together these factors are part of a multi-faceted decision-making process and are balanced to calculate the overall risk/benefit of surgical intervention to the individual patient’s overall quality of life. For instance, a patient’s age was not in itself a contraindication to treatment but was considered alongside other age-related factors that may impact a person’s appropriateness to surgery, recovery and/or potential for complications (e.g., comorbidities) [28, 34, 35, 37, 38, 40–43].

The importance of patients’ experience of pain was discussed in detail across a number of studies [33, 35, 37, 43–46]. Whilst “*pain relief”* or “*pain control*” were described as important, surgical outcomes, the subjectivity and difficulties in quantifying an individual’s pain were acknowledged [35, 37, 44]. Our findings highlight a tendency towards the biomedical model of healthcare and in some cases hesitancy to operate on patients without supporting radiographic or quality of life evidence [37, 44]. This tendency can be problematic in practice, as patients were described as exaggerating pain in the expectation that this would lead to immediate intervention; particularly if a patient “*pushes hard enough*” [46]. Generally, patients were categorised as those wanting: “a *quick fix”* [45], *“‘catastrophisers”’* [46] or “*overly optimistic about their post-surgical outcomes”.* [44] Patients with psychological diagnoses were viewed to experience pain differently. We found evidence that patients with chronic pain syndromes and/or psychological comorbidities might not be offered surgery as their surgical outcomes and potential benefit was unpredictable [44, 46]. Surgeons also described difficulty managing patient expectations, particularly when working in a *“litigious society”* where there is perceived pressure to *“give patients what they want.”* [9, 45].

#### Surgeon demographics

We found little evidence describing the influence of surgeon factors (e.g., location of training, gender, years in practice) on decision making [9, 17, 28, 33]. Bunzli and colleagues, describe how during the COVID-19 pandemic, surgeons were faced with a trade-off between their ethical duty to provide care for patients and their duty of care to themselves and their family. This was particularly apparent for older surgeons and/or those with caring responsibilities who spoke of pressure from family members to stay away from active duty at the hospital during the pandemic [36].

#### Shared decision making

Our synthesis revealed that many surgeons actively seek to involve patients in decisions about their treatment and recovery [33, 35, 36, 45, 47]. However, the realities of achieving shared decision-making were fraught with challenges. This was partly attributed to a perceived mismatch between surgeon and patient priorities surrounding their desired outcomes and treatment preferences [44]. Several studies described the need to manage patient expectations against surgeon treatment preferences, and a patient’s suitability for surgical treatment [36, 38, 40]. This was particularly challenging when referring clinicians (e.g., General Practitioners in the UK) had suggested surgical treatment was needed but this was not deemed appropriate by the surgical team. Surgeons noted that not all patients wish to be involved in decision making. Surgeons broadly categorised patients as: entirely reliant on surgeon recommendation, “*suspicious of surgical recommendation*” or having malleable preferences, which surgeons can alter depending on how treatments are presented [9, 34, 44].

We identified an important role for family and/or social networks when discussing surgical treatments which require significant amounts of post-operative support [33, 34, 37, 42]. For patients from ethnic minority backgrounds, strong cultural hierarchies heightened the role for family members in decision-making [48]. For patients receiving palliative care, what was *“medically best”*, was considered secondary to the end of life goals of patients and family members [33].

### Managerial knowledge – the cost of orthopaedic work

Most studies categorised managerial knowledge as that related to healthcare finance, the cost of orthopaedic provision, and how that impacted on surgical practice. In certain subspecialties, the cost of orthopaedic services (e.g. implants, treatments and/or pharmaceuticals), were an active, conscious drivers of decision making [17, 33]. Similarly, the organisational and/or external regulator (e.g. Orthopaedic Device evaluation panel in the UK) imposed cost and procurement restrictions influencing the implants that can be used [28]. In other studies the cost of an operation was offset by the organisational fees received for doing the procedure [17, 28]. However, we found that pressure to conduct certain procedures to generate hospital revenue encouraged “*overtreatmen*t” and use of expensive equipment even when there is equipoise [28, 33].

We uncovered competition between service managers working to reduce costs, and private organisations (pharmaceutical/device companies) who appeared driven by revenue and share prices [28]. The evidence suggests that placing representatives and marketing campaigns in hospitals, companies could increase surgeons enthusiasm for new technologies and products [17, 28]. We suggest that the extent of influence is tied to the surgeon’s identity. For example, “*Maverick surgeons*” tended to disregard cost concerns and adopted new technology and/or procedures as this reinforced their status as a cutting-edge clinician [17].

### Socialisation of knowledge – community endorsed and community specific evidence

Socialised knowledge reflected the hierarchical nature of orthopaedic surgery and variation in who is considered a trusted source of evidence within the orthopaedic community. Socialised knowledge was inconsistent across included studies, varying within and across specialties, sub-specialties, surgeon identity types, organisations, professional groups, and seniority. Therefore, it was a point of dispersion of knowledge and evidence. We found that evidence only becomes *“legitimate surgical knowledge”* when it is developed and defined by individuals within a surgeon’s specialty or subspecialty (i.e., from within the community) [34]. We found treatment decisions are heavily influenced by the anecdotal experiences and practice of trusted colleagues [14, 17, 28, 35, 49].

Two studies described a strongly embedded elitist community of practice in orthopaedics [9, 17]. This sense of community appears to generate mistrust and dismissal of external evidence and is considered an important driver of decision making with a crucial role in influencing treatment decisions and changing current practice [14, 17, 28, 35]. To counteract this mistrust, specialist societies and key surgical meetings were identified as areas to undertake knowledge sharing and “*good public relations”.* [5] Nevertheless, we found that evidence produced and presented by respected individuals and/or at national societies (e.g., the British Orthopaedic Association, a professional UK-based society) whilst more readily accepted [5], was not considered a sufficient lever for practice change at an individual and organisational level.

Some surgeons were reluctant to work in multi-disciplinary teams and/or accept evidence from those outside of orthopaedics [34], whereas others value team working and collaboration with other professional groups such as physiotherapists [33, 50]. This exception might be explained by organisation and/or sub-specialty norms as surgeons in these studies routinely worked in multi-disciplinary teams [33, 50].

Surgeons described the influence of specialty-specific performance indicators and/or pressure on decision-making. For instance, Emergency Department (ED) doctors in England were considered more likely to offer patients conservative treatment for stable thoracolumbar fractures due to the pressure on discharging patients in EDs to avoid negatively affecting performance targets through admission [9]. Primary care were criticised for recommending patients for surgery earlier than necessary to counterbalance long waiting lists [36], whilst private practice providers were perceived to give little consideration to conservative treatment options [45].

### Cultural normative and political influences

Cultural normative and political influences refer to the wider orthopaedic profession and how elements like national standards, regulation and government policies influenced implementation of evidence [28]. In the absence of formal codified evidence, or when knowledge is ambiguous, surgeons conformed to cultural and professionalised expectations. A paper described “*grey area condition*s” where uncertainty exists around whether surgical or conservative management is ‘best’ [9]. Some surgeons felt pressured by cultural expectations that surgeons operate and “*intervene*” [9]. There was also a perceived threat of medico-legal challenge and judgement from peers and patients if they did not operate [9].

A later study described the highly unstable and uncertain environment during the COVID-19 pandemic where there was a lack of evidence and clinical precedent to work towards [27]. In addition to frequent (almost daily) change, there was significant pressure on resources, with many hospitals working under government mandates to stop, or reduce elective care and move towards a more utilitarian resourcing model where resources were shared for collective good. For *“surgical grey areas”* (e.g., whether sarcoma surgery constituted urgent or elective care) individual surgeons were left to decide patient treatment. In these situations, surgeons relied heavily on their colleagues for an *“added layer of comfort and reassurance”* and worked towards a model of “*least worse decision making”.* [27]

### Training and formal education

We found evidence to suggest that surgical training and education could be described as a “*surgical philosophy”*, where surgeons were *“indoctrinated into a particular approach to practice”* [5]. Surgical education is arguably the foundation of practice and closely tied to implicit knowledge and inclination to trust “*gut feelings*”. Surgeons described a “*cumulative comfort*” from adopting and persisting with practices that were advocated during training. Surgical philosophies promoted during training were well preserved through mentorship and training of junior doctors, and over time became professional, organisational and individual practice norms [5]. Nevertheless, we found little description of the explicit role of training in our included studies. Only one study outlined the “*flexible mobilisation of knowledge*” and showed how, depending on the context, training knowledge may be replaced, or adjusted to new environments as surgeons progress in their careers and move between organisations [5].

## Discussion

Our QES aimed to update and refine an existing conceptual framework to understand and explain *how* drivers of decision-making are applied to orthopaedic surgical work in a dynamic and fluid way. We have described the sources of evidence and knowledge which influence how evidence is viewed and implemented in the day-to-day work of surgeons.

The key finding of our QES is that surgeons appear to adopt a more inclusive approach to evidence based practice which is supported by two decision-making linchpins: surgeon identity, and organisational capacity. These linchpins are the guiding principles of orthopaedic decision making (as described in Fig. 2) which represents a process of medical brokering positioned within their contextual boundaries [30, 31].

We identified that the inter-connected drivers of decision-making are prioritised in real time for each surgical decision. This process plays out in a knowledge and evidence economy where decisions are socialised through the lens of identity, and organisational capacity. These linchpins of surgical practice help to rationalise why the influences on surgical decision-making can fluctuate and vary; and why we observe practice variation. This depiction allows us to understand how the drivers of decision-making are differentially weighted depending on the context. Our reconceptualised portrayal rejects the siloed presentation of the evidence and knowledge sources that were identified as drivers of decision-making in orthopaedics in the original review in favour of a more fluid approach [4].

We found limited support for formal codified knowledge (such as clinical guidelines) as the key driver of orthopaedic surgical decision-making. Our findings revealed a strong preference towards implicit, experiential knowledge which cannot be legitimately codified and easily regulated. This goes someway to explain why these categories of evidence fall lower in the practice evidence hierarchy, when socialised knowledge is privileged. The failure to capture and account for the power attached to the tacit knowledge of surgeons illuminates why formal codified knowledge is often rendered inappropriate for orthopaedic surgical work. For example, why RCTs continue to fail to be implemented in orthopaedics despite the growth in research funding and positive exemplars of practice change [3, 13–16, 39]. Surgeon identity and the place a surgeon works may partially explain why, discontinuation rates [51, 52] and recruitment challenges remain markedly higher in surgical trials when compared to other medical specialties [53]. However, we are observing recent progress in delivering high quality research evidence [5, 13–17] which demonstrates that profession-wide cultural resistance does not exist in orthopaedics.

We have previously published the “vicious cycle of uncertainty” in orthopaedics [9]. Where a lack of evidence is used to justify the need for high quality evidence (i.e. an RCT), but the creation of this evidence is prohibited by surgeons’ reliance on informal knowledge. In the context of a trial this *vicious cycle* manifests as inability to generate equipoise and poor trial recruitment [9, 54]. The data from our review suggests that the intersection between surgical decision-making, equipoise and research feasibility (i.e. the possibility of successfully engaging the clinical community to deliver a trial) warrants further exploration as it is not unilaterally experienced across the specialty.

For example, for divisive research questions where we compare interventions or techniques that reflect a surgeon’s craft or skill (i.e. surgeon identity), it is essential to consider whether an RCT is deliverable. We suggest for divisive questions – dependent of skill, we need a collective mindset shift as surgical research communities, to consider alternative methodological approaches. There is a trade-off to be had between (i) funding a trial because it may provide the level of evidence required to change surgical practice versus (ii) the risk of delivering unfeasible or underpowered studies resulting in no evidence generation. However, alternative approaches need to balance the commissioning of lower-level studies that are potentially feasible – that may also be insufficient to initiative practice change. It is important to review recent success stories in orthopaedic research to identify what evidence (including but not exclusive to trials) has been delivered successfully and those studies that have generated real practice change.

To improve EBP, we therefore need to focus improvement efforts at both ends of the research pathway – evidence creation and evidence implementation. A cultural shift in how we privilege scientific evidence must evolve from within the surgical community for it to be legitimised within the profession. Therefore, the drive to change needs to begin from within. We propose that the research and surgical community co-produce future research and implementation strategies that are tailored to surgery and are designed to overcome clinical uncertainty – our findings show we need to avoid research which “threaten” a surgeon’s identity and craft. We need to work in partnership with the surgical community to ascertain what evidence they *value* and *need* to inform their practice so that they are able to place a higher value upon evidence. Strategies are required which help to promote a cultural shift towards using evidence (where it is available) and a move away from an overreliance on anecdotal and experiential knowledge sources [9]. This can only happen if the evidence we are producing is clinically meaningful and perceived as useful. Our findings demonstrate that, surgeons feel it is important to tailor their decisions to the individual needs of the patient in front of them (the personality of the fracture). As such, we need to ensure that research funders and academics work to create methodologically innovative and well-resourced solutions to ensure that the evidence we are producing is inclusive of this tailoring by design. This change in approach will be fundamental, to facilitating the adoption of trial evidence into practice, which is often criticised for not being applicable to “real-world” patients [9, 55].

We know on-going work aims to encourage evidence-based leadership interventions to drive improvement from within orthopaedics [56, 57]. We recognise the potential of professional societies and networks (e.g. British Orthopaedic Association in the UK), to standardise and disseminate evidence-based guidelines. Whilst we identified strong support for this approach in our review, its sustainability and spread has not been captured in the literature. Greater methodological rigour and transparency in the process for producing society specific guidelines and standards would be required if adopting this approach. Our framework positions a more inclusive approach to EBP in surgery. We position a series of fluid, competing and nonlinear context-specific factors, which prevent “*discrete improvement projects to implement scientific evidence*” from being effective in orthopaedics [58]. Research in this field needs to move towards multifaceted and evidence-based implementation strategies which simultaneously target several of the knowledge and evidence sources that we have identified. We position surgical decision-making not as a process or singular construct but as something requiring complex intervention. An initial step is the use of implementation science and implementation theory (e.g., The Consolidated Framework for Implementation Research) [59] to encourage innovation in implementation science and the development of implementation strategies that can be used to accurately measure practice change [58]. For this approach to be a success, radical change in how evidence is viewed, created, and implemented in orthopaedics is required. The challenge ahead is for researchers and surgeons to work together to co-create innovative research methods and implementation strategies, that are designed specifically to foster a culture of EBP.

### Strengths and limitations

We found no obvious patterns within our data to suggest that the drivers of decision-making vary internationally, suggesting that the review could be used to inform our understanding of orthopaedic surgical decision-making on a global scale. However, it is important to note that our included studies primarily represented middle-or high-income countries and that data pertaining to the cost of orthopaedic work was limited. Greater exploration of the influence of resourcing constraints and public versus private health systems warrants further exploration. Further key strengths of our QES include our robust search strategy and our processes for screening, data extraction and quality appraisal.

Five of our included studies were led by members of our authorship team AG [3, 5, 17, 28] and AS [9], which might be considered a limitation. The original mixed method review has been instrumental in changing the way that we view decision-making, and to account for this, we sought independence at key stages of the review wherever possible. For example, screening and data extraction were undertaken independently by KJ and AS. AG was not involved in analysis until the final stages of interpretation. All authors are non-clinical academics with experience of, and a particular interest in orthopaedic surgery research, trials and qualitative methodology and therefore hold a bias towards EBP. We are not orthopaedic surgeons but work closely with academic and practising surgeons. Most of our included studies used interview methods (*n* = 23, 92%) and thematic analysis (*n* = 19, 76%), future qualitative research would benefit from using a range of qualitative methodologies. Observations may, through watching decision-making in practice help inform the design of implementation strategies and innovative research methodologies.

Our re-conceptualisation of orthopaedic decision making, is a representation of the international qualitative evidence that exists on this topic (i.e. it is not the product of a single qualitative data set). Whilst, this is a key strength of our framework, our understanding of what drives decision-making is limited to the data that has been reported within our included studies. This is as an area which requires greater exploration, as there are some drivers for which little data was identified for example, the cost of orthopaedic work, training and education, which are inherently complex.

## Conclusion

This QES identified key sources of evidence and knowledge that have been consistently applied as driving orthopaedic decision-making. We have expanded an existing conceptual framework to go beyond identifying *what* factors influence decision-making in orthopaedics to present an explanatory framework to describe *how* these drivers constantly compete and interact during surgical decisions. The findings suggest that orthopaedic decision-making is routinely in a state of flux, and it is dependent on the real-time context of surgical decisions which generates limited and an inconsistent role of clinical evidence in decision-making. Two mediating linchpins of surgeon identity and organisational capacity enacted through a socialisation of knowledge generate a more inclusive surgeon derived approach to EBP. Academics and surgeons must now work to identify what evidence (in the most broadest sense) is required and valued to underpin orthopaedic practice to shift the orthopaedic profession away from relying on informal, experiential knowledge towards EBP. In doing so we need to view orthopaedic decision-making as a complex intervention which requires multi-faceted, evidence-based implementation strategies that are developed in partnership with the surgical community to drive a change in how scientific evidence is perceived and used in their work.

## Electronic supplementary material

Below is the link to the electronic supplementary material.

Supplementary Material 1: Characteristics of included studies

Supplementary Material 2: PRISMA 2020 checklist

Supplementary Material 3: Search strategy

Supplementary Material 4: Quality appraisal for included studies using CASP tool

Supplementary Material 5: Exemplar quotes for thematic synthesis

Supplementary Material 6: Conceptual Framework of the sources of evidence and drivers of variation in orthopaedic surgical work (Grove et al., 2016 versus. Conceptual Framework of Evidence-Based Orthopaedics: The Linchpin Model

Supplementary Material 7: Patient related factors

## Data Availability

No datasets were generated or analysed during the current study.

## Abbreviations

CASP: Critical Appraisal Skills Programme
ED: Emergency Department
ENTREQ: Enhancing Transparency in Reporting the synthesis of qualitative research
EBP: Evidence Based Practice
QES: Qualitative Evidence Synthesis
RCT: Randomised Controlled Trial
UK: United Kingdom
