# Supplementary material for: Can we ever have evidence-based decision making in orthopaedics? A qualitative evidence synthesis and conceptual framework
Source: BMC Med Inform Decis Mak. 2025 Jul 1;25:216. doi: 10.1186/s12911-025-03032-5 (PMC12211141; doi:10.1186/s12911-025-03032-5)
Supplement: Supplementary file 1 — Supplementary Material 1: Characteristics of included studies [file 12911_2025_3032_MOESM1_ESM.pdf]

Additional file 1 Characteristics of included studies

Reference (year)	Country (Setting*)	Study design<br><br>(Total n and by stakeholder group)	Participant characteristics	Orthopaedic procedure(s)	Study objectives	Method of analysis*
Adogwa et al., (2021)	U.S (Hospital, single site)	Qualitative interview study<br>(n=11 n=6 patients, n=5 spine surgeons)	Years of experience: average years in practice 5.<br><br>Training level (grade), age, gender and ethnicity not reported.	Surgery for correction of adult spinal deformity	To understand patients' and spine surgeons' perspectives about shared decision-making around surgery for adult spinal deformity	Constant comparison method. Axial coding used to identify emergent themes and decisional needs.
Baker et al (2019)	US (Hospital multi-site)	Qualitative interview study<br><br>(n=2, 9 orthopaedic surgeons, 3 hospitalists, 3 geriatricians, 5 nurses, 3 occupational therapists, 3 physical therapists and 2 clinical ethicists.)	Gender: 14 Females, 11 Males, 3 unspecified.<br><br>Training level (grade), age and ethnicity not reported.	Surgical interventions for patients with hip fractures who are hospitalised with dementia	To determine the factors that members of a typical geriatric interprofessional team consider when deciding on surgical intervention for patients with hip fractures and dementia	Inductive and deductive analysis of the content of verbatim transcripts. Once coding was complete they developed summaries for each theme
Barton et al (2021)	US ("Physicians from across North America")	Qualitative study consisting of focus groups and/or interviews<br><br>(n=14 orthopaedic surgery n=6; neurosurgery n=5, radiation oncology n=2 and physiatry n=1)	Training level (grade): All faculty levels from instructor to professor.<br><br>Gender: 11 Male, 3 Female<br><br>Age and ethnicity not reported.	Operative and non-operative management for spinal metastatic disease	To evaluate the decision-making process for treatment of spinal metastases from the clinician's perspective.	Thematic analysis (Braun and Clark 2006).
Brown et al (2018)	South Africa (Public tertiary teaching hospital in a unit dedicated to the treatment of	Qualitative focus group study<br><br>(n=23 orthopaedic consultants and registrars 9; registered nurses from orthopaedic wards, clinics	Training level (grade): see study design column<br><br>Ethnicity: White 13, Indian 4, Africal (isiZulu) 4, Mixed race 2.	Treatment of osteosarcoma	To identify the cultural factors associated with discussing the different treatment options of osteosarcoma – and to explore healthcare professionals' responses to	Thematic analysis (Braun and Clark 2006)

	musculoskeletal tumours in a largely rural province of Kwazulu-natal South Africa)	and pain service 5, allied health professionals (physiotherapists, occupational therapists, dieticians, social worker) 9).	Gender: 15 Females, 8 Males.<br><br>Age and years experience not reported.		these cultural factors – from the healthcare professionals' perspective	
Bunzil et al (2017)	Australia (Single, tertiary teaching hospital)	Qualitative interview study<br><br>(n=20 orthopaedic surgeons)	Training level (grade): 15 consultants, 5 registrars.<br><br>Years experience: of performing Total Knee Arthroplasty (TKA) ranged from 6 months to 30 years.<br><br>Age, gender, and ethnicity not reported.	Surgery for total knee arthroplasty	To explore the barriers and facilitators to decision aid uptake among orthopaedic surgeons	Adopting a three stage implementation approach to analysis using the Theoretical Domains Framework. Frequencies of belief produced to provide a range of interview responses and to identify how interviews correspond to the TDF.
Bunzil et al (2021)	Public and private hospitals in 14 countries.	Qualitative interviews<br><br>(n=18 specialist sarcoma surgeons)	Years experience: average 19 years as a sarcoma surgeon.<br><br>Training level (grade), age, gender and ethnicity not reported.	Orthopaedic oncology surgeries	To explore hat decisions to sarcoma surgeons have to make along the patient journey during COVID-19 and what cues (i.e. relevant items of information and rules) are associated with each decision)	Inductive thematic analysis
Coole et al (2021)	England (NHS Hospital multi-site)	Qualitative study 19 group interviews were conducted with between 2 and 4 participants, remainder were individual interviews<br><br>(n=40 12 allied health professionals, nurses 95 Occupational Therapists, 4	Training level (grade): surgeons: 12 consultants, GPs: 14 partners, 1 salaried, 1 registrar, AHPs and Nurses: Band 7, 8, Band 6, 3, Band 5, 1.<br><br>Years experience: 1-32 years.<br><br>Male: 26, Female: 14	Treatment of total knee replacement and total hip replacement	To explore the views and experiences of clinicians in treating working patients undergoing total hip or knee replacement.	Framework methodology and thematically

		Physiotherapists and 3 nurses), 12 orthopaedic surgeons and 16 General Practitioners).	Age and ethnicity not reported.			
Dismore et al (2021)	UK (NHS hospital single site)	Qualitative interview study<br><br>(n=14 orthopaedic practitioners)	Training level (grade): 11 consultants, 3 registrars.<br><br>Years experience: 3-25 years of orthopaedics<br><br>Age: range 29-52 years<br><br>Female: 2, Male 12<br><br>Ethnicity not reported	Forefoot surgery for hallux valgus (bunion) or hallux rigidus (arthritis of the big toe joint)	Orthopaedic surgeons' experiences of providing care, their decision-making process for surgery, their views of PC and use of behavioural support in surgical practice using a qualitative approach.	Thematic analysis (Braun and Clarke)
Frankel et al (2016)	Canada (Community hospital and academic centre – academic and urban centres)	Qualitative interview study<br><br>(n=14 orthopaedic surgeons)	Male 12, Female 2<br><br>Age: <50 7; 51-64 6, 65+ 1<br><br>Training level (Grade), years experience and ethnicity not reported.	Total joint arthroplasty	To elucidate surgeons' perspectives on appropriateness for total joint arthroplasty (TJA) - to inform development of a TJA surgeon patient decision support tool.	Analytical steps guided by Giorgi's procedures/ Each coder independently read and re-read three transcripts and developed meaning units (codes) relevant to the objective. They met to discuss and reach consensus on the relevant themes.
Grove et al (2018)	UK (3 English NHS hospitals)	In depth multi-method qualitative case studies consisting of observations of day-to-day practice at each of the three case sites, interviews with NHS staff	Training level (grade), age, gender, ethnicity and years experience not reported.	Total hip replacement for end-stage arthritis	To identify where, when and how evidence and knowledge are used in healthcare decision-making and how variation in these factors contributes to different approaches to implementation of clinical guidance in practice.	Data were analysed, integrated and triangulated within cases before comparative case analysis. Thematic analysis of each case site. Data was integrated using the

		(clinical 34, allied health professionals, 17 managers and national stakeholders 13) (n=64) supplementary documents (n=121)				Pillar Integration Process.
Grove et al (2020)	England (Three NHS hospitals in England.)	A comparative case study consisting of n=64 clinical, allied health professionals, managers) and national stakeholders interviews and observations of orthopaedic departments for a minimum of three months at each of the three case sites and documentary analysis<br><br>(clinical 34, allied health professionals, 17 managers and national stakeholders 13) (n=64) supplementary documents (n=121)	Training level (grade), age, gender, ethnicity and years experience not reported..	Orthopaedic surgery.	To explore and describe how professionalised groups of surgeons, at the forefront of service delivery, mobilise knowledge in practice.	Cross-case multi-level analysis - followed the stages of data familiarisation, coding and development of categories from codes (Bernard & Ryan. 2010).<br>Analysis of cases structured using four levels of analysis described by Ferlie and Shortell (2001).<br>Data analysed across the three cases using the roadmap method with divergent techniques (Eisenhardt 1989).
Grove et al (2021)	England (Three NHS hospitals in England.)	A comparative case study<br><br>(clinical 34, allied health professionals, 17 managers and national stakeholders 13) (n=64) supplementary documents (n=121)	Training level (grade), age, gender, ethnicity and years experience not reported.	Hip arthroplasty	To present a typology of orthopaedic surgeons' professional identities and through abductive analysis, show the ways in which identity work serves a purpose: as a rhetorical device to enhance legitimacy and signify position; as a means for self-stratification in relation to	Abductive thematic approach (Braun and Clarke 2006). To develop a conceptual understanding of the empirical data and produce theoretical ideas followed abductive analysis (Timmermand and Tavory 2012).

					others; and as a predictor of adherence to Evidence Based Practice.	Comparative cross-case analysis to look for similarities, contrasts and anomalies between the hospitals, surgeons and their enactment of EBP using Eisenhardts case study road map (1989)
Haider et al (2020)	England (Hospital based trauma and orthopaedic departments)	Online questionnaire with free text responses<br><br>(n=113 regional trainee representatives of the British Orthopaedic Trainees Association)	Training level (grade): See study design column<br><br>Age, gender, ethnicity and years experience not reported.	Trauma meetings in orthopaedic surgery	The study aims to reveal what trainees perceive they are currently learning,<br>the positive and negative educational aspects of the trauma meetings and trainee recommended improvements to maximise trainee learning.	Thematic analysis (Braun and Clarke)
Hsu et al (2017)	USA ("Orthopaedic providers")	Longitudinal qualitative interview study. Interviews were conducted during the first year of decision-aid implementation and at one year follow-up<br>(n=19 orthopaedic and cardiology clinicians)	M: 15 F: 4 (Year 1) M: 11, F: 4 (Year 2)<br><br>Training level (grade), age, ethnicity and years experience not reported.	Implementation of decision aid in cardiology and orthopaedics	The evaluation captured qualitative information on the implementation process in six specialties and in-depth provider reactions to the project (wider project evaluating the effect of decision aid distribution on health care utilisation and care costs for preference-sensitive conditions related to elective surgery) in two specialties. In addition to the implementation processes, the data	Thematic approach. Initial findings mapped to the four stages of communication described as necessary for patient centred care in the conceptual model of Halley et al.

					documented the attitudes, reactions and experiences of frontline providers who distributed the Decision Aids and engaged in Shared Decision Making with patients.	
Jefferson et al (2017)	UK (NHS, major trauma centre or equivalent tertiary hospital, trauma unit, or secondary care hospital, district hospital or other)	Online questionnaire with free text responses conducted as part of a Randomised Controlled Trial<br><br>(n=265 surgeon members of the British orthopaedic association and British elbow and shoulder society)	Training level (grade): consultant: 218; speciality trainee ST7 or ST8 18, Speciality trainee ST1 or ST6 11, staff associate specialists 10, fellows 6, missing 1.<br><br>Years experience of treating fractures of the proximal humerus: 0-5 years 25; 6-10 years 82; 11-15 years; 57; 16-20 years 41 >21 60.<br><br>Male 249, Female 16<br><br>Age: <35: 23, 36-45 98, 46-55 88, 56-65 54 >66 2.<br><br>Ethnicity not reported.	Surgical and non-surgical treatment for adults with displaced proximal humerus fractures involving the surgical neck	To explore whether orthopaedic surgeons have adopted the proximal Fracture of the Humerus: evaluation by Randomisation (pRoFHeR) trial results routinely into clinical practice	Free text responses were analysed using Framework analysis
Madsen et al (2021)	Denmark (Orthopaedic outpatient shoulder clinic, Silkeborg regional hospital)	Mixed methods study. An Interrater agreement study and a qualitative study with two focus group interviews<br><br>(n=7 3 extended scope physiotherapists [ESP] and 4 orthopaedic surgeons [OS])	Training level (grade): consultant 3 and registrar 1. Physiotherapist grade not reported.<br><br>Years experience: all had more than 3 years of experience at the shoulder clinic, Silkeborg hospital.	Diagnosis and treatment for patients with shoulder disorders	1a) To evaluate agreement on diagnosis between ESPs and OSs examining patients with shoulder disorders 1b) To evaluate agreement on treatment plan between ESPs and OSs examining patients with shoulder disorders (primary aim) 2) To explore and evaluate the	Thematic analysis (Braun and Clarke, ?date unspecified?)

			Age, gender and ethnicity not reported.		inter-professional collaboration between ESPs and OSs. Qualitative component: to gain knowledge about staff interpretations, experiences and opinions on interprofessional collaboration.	
Moore et al (2017)	England and Wales (5 high volume NHS orthopaedic departments)	Qualitative interview study<br><br>(n=12 orthopaedic surgeons)	Training level (grade): all consultants<br><br>Years experience: average 14 years treating prosthetic joint infection.<br><br>Age: Average 49 years.<br><br>Gender: All male<br><br>Ethnicity not reported.	Revision surgery for Prosthetic joint infection after hip arthroplasty.	To characterise consultant orthopaedic surgeons' decisions about performing either one-stage or two-stage revision surgery for patients with deep prosthetic infection (PJI) after hip arthroplasty, and to identify whether a randomised trial comparing one-stage with two-stage revision would be feasible	Thematic analysis (Braun and Clarke 2006)
Phelps et al (2019)	England (7 NHS hospitals)	Qualitative interview study conducted as part of a mixed methods process evaluation for a Randomised Controlled Trial	Training level (grade), age, gender, ethnicity and years experience not reported.	Intradeullary nails versus distal locking plates for fractures of the distal femur	Patients' experiences of participating and staff experiences of being involved in an orthopaedic trauma trial: TrAFFix	Inductive with themes derived from the data.
Rath et al (2017)	India (major public tertiary care hospitals)	Multi-methods observational study, key informant interviews and four focus groups with health professionals.<br><br>(n=35, 24 surgeons and research associates, 9	(n=11 Interviews and four focus groups (n= not reported) with key informants, clinical leads, residents and nursing staff from orthopaedics, anaesthesia, geriatrics, medicine and physiotherapy.	Hip fractures in older adults	To document current practices and identify barriers and facilitators to adopting best practice guidelines and recommend improvements in the management of older adults with hip fractures in Delhi India	Thematic analysis

		patients and 2 personal consultees)	Training level (grade), age, gender, ethnicity and years experience not reported.			
Rehman et al (2019)	Canada (Neurosurgical practices n=6 Ontario)	Qualitative interview study<br><br>n=18 (12 patients, 6 neurosurgeons)	Years experience: range 8-26<br><br>Age: Range 45-68 years<br><br>Training level (grade), gender and ethnicity not reported.	Risks and benefits of lumbar decompressive surgery (LDS) for sciatica	To explore sciatica patients' preoperative expectations and<br>their spine surgeon's perspectives with regards to understanding regarding LDS, postoperative outcomes, and information required for informed decision-making.	"Inductive content analysis and thematic analysis?"
Robba et al (2019)	UK (Consultant hand surgeons UK-wide)	Qualitative interview study<br><br>n=10 hand surgeons	Training level (grade): consultants.<br><br>Age, gender, ethnicity and years experience not reported.	Treatment of triangular fibrocartilage complex (TFCC)injuries	To explore the perceptions and experiences of consultant wrist surgeons managing TFCC injuries, with the purpose of understanding the factors informing 'expert' clinical decision-making. This might help to explain existing variations in TFCC management, guide future research and inform clinical care	Thematic analysis (Braun and Clarke 2006)
Scantlebury et al (2022)	UK hospitals (n=13)	Qualitative interviews with surgeons conducted as part of a mixed methods randomised pilot study<br><br>N= 19 spinal surgeons.	Training level (grade): consultants and spinal fellows.<br><br>Male: 14 Female: 5<br><br>Age, ethnicity and years experience not reported.	Stable thoracolumbar fractures without spinal cord injury	What influences surgical decision making for the treatment of stable thoracolumbar fractures in the UK and the implication of variation in spinal surgical work on the creation and adoption of future evidence.	Thematic analysis (Braun and Clarke 2006)

Schmidtke et al (2022)	Acute hospitals in England	Mixed methods study. Qualitative interviews surgeons and health professionals from multiple medical and surgical specialties<br>N= 25 academics: 6, surgeons: 9, other 23 (dietician, speech and language therapists, radiologist, gastroenterologist, general practitioner) involved in six surgical trials in stroke, gastro-oesophageal reflux disease, abdominal aortic aneurysm, knee replacement, varicose veins (2 trials).	Training level (grade): not reported.<br><br>Years experience: median 20<br><br>Age, gender and ethnicity not reported.	KAT trial – patella resurfacing	Examine the uptake of six trials that produced actionable findings to describe the effects of evidence on practice and the reasons for those effects.	Deductive and inductive coding using the CFIR as a analytical framework
Shaw et al (2022)	UK hospitals undertaking major orthopaedic, colorectal and/or cardiac surgery	Multi-method qualitative video-recordings of pre-operative consultations, longitudinal interviews and focus groups<br><br>(n=31 patients, 19 relatives and 37 surgeons and anaesthetists (21) representing orthopaedics (n=3), colorectal (8) and/or cardiac (3)surgery)	Training level (grade): Not reported<br><br>Years experience, age, gender and ethnicity not reported.	High risk patients offered major surgery. Orthopaedic patients (hip and knee)	When and why clinicians and patients can share decision-making about major surgery	Thematic analysis and constant comparison
Sutton et al (2021)	Australia (Urban and rural GP and orthopaedic	Qualitative interview study	Training level (grade): not reported.<br><br>Age: range 33-62	Conservative and surgical management	To 1) explore the attitudes towards and understanding of osteoarthritis), and 2) gain a deeper understanding	Thematic analysis Braun and Clarke 2006)

	practices Tasmania)	(n=27) General Practitioners (17) and orthopaedic surgeons (10)	Gender: 4 GPs and all surgeons were male.<br><br>Years experience and ethnicity not reported.	for osteoarthritis	of conservative and surgical management and 3) identify key barriers and challenges.	

n, Number; NICE, National Institute for Health and Care Excellence; NHS, National Health Service; GP, General Practitioner

*As described by study authors
