# Supplementary material for: Can we ever have evidence-based decision making in orthopaedics? A qualitative evidence synthesis and conceptual framework
Source: BMC Med Inform Decis Mak. 2025 Jul 1;25:216. doi: 10.1186/s12911-025-03032-5 (PMC12211141; doi:10.1186/s12911-025-03032-5)
Supplement: Supplementary file 3 — Supplementary Material 3: Search strategy [file 12911_2025_3032_MOESM3_ESM.pdf]

Additional file 3 Search strategies

All databases were first searched on 19th January 2022. The strategies reported below are those from the most recent searches on 21st March 2023.

MEDLINE ALL
(includes: Epub Ahead of Print, In-Process & Other Non-Indexed Citations, Ovid MEDLINE Daily and Ovid MEDLINE)
via Ovid http://ovidsp.ovid.com/
Date range: 1946 to March 20, 2023
Date searched: 21st March 2023
Records retrieved: 4169

1 Orthopedics/ (23676)
2 Orthopedic Surgeons/ (1259)
3 orthop?edi$.ti,ab. (104077)
4 ((hand or shoulder or elbow or wrist) adj surgeon$).ti,ab. (5138)
5 ((spine or spinal or hip or knee or ankle or foot) adj surgeon$).ti,ab. (4509)
6 1 or 2 or 3 or 4 or 5 (120668)
7 Physical Therapists/ (3025)
8 Podiatry/ (2393)
9 (physical therapist$ or physiotherapist$).ti,ab. (18000)
10 hand therapist$.ti,ab. (409)
11 podiatrist$.ti,ab. (1032)
12 7 or 8 or 9 or 10 or 11 (21834)
13 (surgery or surgeries or surgical$ or operation$ or operate$ or operative$).mp. (4004589)
14 12 and 13 (3437)
15 6 or 14 (123388)
16 Decision Making/ (104078)
17 exp Clinical Decision-Making/ (15099)
18 Decision Making, Shared/ (1839)
19 (decision$ adj2 (making or make? or made or maker?)).ti,ab. (230461)
20 (decision$ adj3 (clinical or evidence or knowledge or practice$ or procedure$ or surgeon$ or surgery or surgeries or surgical$ or operation$ or operate$ or operative$ or treatment$ or technique$ or manag$ or care)).ti,ab. (126345)
21 (decision$ adj2 (share$ or sharing)).ti,ab. (13909)
22 16 or 17 or 18 or 19 or 20 or 21 (354585)
23 15 and 22 (3336)
24 Practice Patterns, Physicians/ (66739)
25 Practice Patterns, Nurses'/ (2961)
26 Professional Practice/ (17095)
27 Professional Autonomy/ (9703)
28 ((variation$ or vary$ or varies or varied$ or variability$) adj3 (practice$ or procedure$ or surgeon$ or surgery or surgeries or surgical$ or operation$ or operate$ or operative$ or treatment$ or technique$ or manag$ or care)).ti,ab. (53221)
29 ((variation$ or vary$ or varies or varied$ or variability$) adj2 (local$ or regional$ or national$ or international$ or country or countries or geographic$)).ti,ab. (37036)
30 (practice$ adj2 (pattern$ or trend$)).ti,ab. (12253)
31 24 or 25 or 26 or 27 or 28 or 29 or 30 (186457)
32 15 and 31 (3007)
33 23 or 32 (6041)

34 Evidence-Based Practice/ (11918)
35 Evidence-Based Medicine/ (75994)
36 Evidence-Based Nursing/ (4062)
37 ((evidence-based or evidencebased) adj (practice$ or medicine or nursing or treatment$ or surgery or care)).ti,ab. (40646)
38 ((evidence-based or evidencebased) adj2 (p?ediatric$ or child health)).ti,ab. (243)
39 or/34-38 (115572)
40 15 and 39 (1336)
41 33 or 40 (7097)
42 (decision$ adj3 orthop?edi$).ti,ab. (191)
43 ((variation$ or vary$ or varies or varied$ or variability$) adj3 orthop?edi$).ti,ab. (229)
44 ((evidence-based or evidencebased) adj orthop?edi$).ti,ab. (64)
45 42 or 43 or 44 (482)
46 41 or 45 (7257)
47 limit 46 to yr="2014 -Current" (4367)
48 exp animals/ not humans.sh. (5104362)
49 47 not 48 (4342)
50 limit 49 to english language (4169)

Key:
/ = subject heading (MeSH heading)
sh = subject heading (MeSH heading)
exp = exploded subject heading (MeSH heading)
$ = truncation
? = optional wild card character - stands for zero or one character
ti,ab = terms in title or abstract fields
mp = terms in title, abstract, subject heading, or original title fields
adj3 = terms within three words of each other (any order)
adj = terms next to each other (in order specified)

Embase
via Ovid http://ovidsp.ovid.com/
Date range: 1974 to 2023 March 20
Date searched: 21st March 2023
Records retrieved: 6202

1 orthopedics/ (25141)
2 orthopedic surgeon/ (11201)
3 exp orthopedic specialist/ (1582)
4 orthopedic nursing/ (1153)
5 pediatric orthopedic surgeon/ (3)
6 orthop?edi$.ti,ab. (141137)
7 ((hand or shoulder or elbow or wrist) adj surgeon$).ti,ab. (5777)
8 ((spine or spinal or hip or knee or ankle or foot) adj surgeon$).ti,ab. (7135)
9 or/1-8 (164186)
10 physiotherapist/ (27081)
11 physiotherapist assistant/ (108)
12 podiatrist/ (929)
13 (physical therapist$ or physiotherapist$).ti,ab. (31724)
14 hand therapist$.ti,ab. (534)
15 podiatrist$.ti,ab. (1455)

16 or/10-15 (40127)
17 (surgery or surgeries or surgical$ or operation$ or operate$ or operative$).mp. (5373616)
18 16 and 17 (6653)
19 9 or 18 (169528)
20 decision making/ (277760)
21 clinical decision making/ (64970)
22 medical decision making/ (93300)
23 shared decision making/ (14260)
24 (decision$ adj2 (making or make? or made or maker?)).ti,ab. (322792)
25 (decision$ adj3 (clinical or evidence or knowledge or practice$ or procedure$ or surgeon$ or surgery or surgeries or surgical$ or operation$ or operate$ or operative$ or treatment$ or technique$ or manag$ or care)).ti,ab. (188693)
26 (decision$ adj2 (share$ or sharing)).ti,ab. (20601)
27 20 or 21 or 22 or 23 or 24 or 25 or 26 (668926)
28 19 and 27 (6035)
29 clinical practice/ (355124)
30 nursing practice/ (6024)
31 professional practice/ (57704)
32 ((variation$ or vary$ or varies or varied$ or variability$) adj3 (practice$ or procedure$ or surgeon$ or surgery or surgeries or surgical$ or operation$ or operate$ or operative$ or treatment$ or technique$ or manag$ or care)).ti,ab. (79266)
33 ((variation$ or vary$ or varies or varied$ or variability$) adj2 (local$ or regional$ or national$ or international$ or country or countries or geographic$)).ti,ab. (46778)
34 (practice$ adj2 (pattern$ or trend$)).ti,ab. (19677)
35 29 or 30 or 31 or 32 or 33 or 34 (544045)
36 19 and 35 (6214)
37 28 or 36 (11519)
38 evidence based practice/ (81194)
39 evidence based medicine/ (123290)
40 evidence based nursing/ (4701)
41 ((evidence-based or evidencebased) adj (practice$ or medicine or nursing or treatment$ or surgery or care)).ti,ab. (53112)
42 ((evidence-based or evidencebased) adj2 (p?ediatric$ or child health)).ti,ab. (362)
43 38 or 39 or 40 or 41 or 42 (231338)
44 19 and 43 (2556)
45 37 or 44 (13346)
46 (decision$ adj3 orthop?edi$).ti,ab. (228)
47 ((variation$ or vary$ or varies or varied$ or variability$) adj3 orthop?edi$).ti,ab. (287)
48 ((evidence-based or evidencebased) adj orthop?edi$).ti,ab. (65)
49 46 or 47 or 48 (576)
50 45 or 49 (13513)
51 limit 50 to yr="2014 -Current" (8279)
52 limit 51 to english language (8081)
53 limit 52 to conference abstracts (1819)
54 52 not 53 (6262)
55 (animal/ or animal experiment/ or animal model/ or animal tissue/ or nonhuman/) not exp human/ (6764890)
56 54 not 55 (6202)

Key:
/ = subject heading (Emtree heading)

exp = exploded subject heading (Emtree heading)
$ = truncation
? = optional wild card character - stands for zero or one character
ti,ab = terms in title or abstract fields
mp = terms in title, abstract, subject heading, or original title fields
adj3 = terms within three words of each other (any order)
adj = terms next to each other (in order specified)

PsycINFO
via Ovid http://ovidsp.ovid.com/
Date range: 1802 to March Week 2 2023
Date searched: 21st March 2023
Records retrieved: 117

1 orthop?edi$.ti,ab,id. (3414)
2 ((hand or shoulder or elbow or wrist) adj surgeon$).ti,ab,id. (23)
3 ((spine or spinal or hip or knee or ankle or foot) adj surgeon$).ti,ab,id. (18)
4 1 or 2 or 3 (3448)
5 physical therapists/ (691)
6 (physical therapist$ or physiotherapist$).ti,ab,id. (3021)
7 hand therapist$.ti,ab,id. (21)
8 podiatrist$.ti,ab,id. (58)
9 5 or 6 or 7 or 8 (3155)
10 (surgery or surgeries or surgical$ or operation$ or operate$ or operative$).mp. (182812)
11 9 and 10 (148)
12 4 or 11 (3576)
13 decision making/ (85892)
14 (decision$ adj2 (making or make? or made or maker?)).ti,ab,id. (140350)
15 (decision$ adj3 (clinical or evidence or knowledge or practice$ or procedure$ or surgeon$ or surgery or surgeries or surgical$ or operation$ or operate$ or operative$ or treatment$ or technique$ or manag$ or care)).ti,ab,id. (29389)
16 (decision$ adj2 (share$ or sharing)).ti,ab,id. (4367)
17 13 or 14 or 15 or 16 (164788)
18 12 and 17 (158)
19 ((variation$ or vary$ or varies or varied$ or variability$) adj3 (practice$ or procedure$ or surgeon$ or surgery or surgeries or surgical$ or operation$ or operate$ or operative$ or treatment$ or technique$ or manag$ or care)).ti,ab,id. (9456)
20 ((variation$ or vary$ or varies or varied$ or variability$) adj2 (local$ or regional$ or national$ or international$ or country or countries or geographic$)).ti,ab. (5918)
21 (practice$ adj2 (pattern$ or trend$)).ti,ab. (1717)
22 19 or 20 or 21 (16780)
23 12 and 22 (38)
24 evidence based practice/ (20497)
25 (evidence-based adj (practice$ or medicine or nursing or treatment$ or surgery or care)).ti,ab,id. (22687)
26 (evidencebased adj (practice$ or medicine or nursing or treatment$ or surgery or care)).ti,ab,id. (32)
27 ((evidence-based or evidencebased) adj2 (p?ediatric$ or child health)).ti,ab,id. (52)
28 24 or 25 or 26 or 27 (30742)
29 12 and 28 (39)
30 (decision$ adj3 orthop?edi$).ti,ab,id. (9)

31 ((variation$ or vary$ or varies or varied$ or variability$) adj3 orthop?edi$).ti,ab,id. (5)
32 ((evidence-based or evidencebased) adj orthop?edi$).ti,ab,id. (0)
33 30 or 31 or 32 (14)
34 18 or 23 or 29 or 33 (221)
35 limit 34 to yr="2014 -Current" (127)
36 limit 35 to english language (117)

Key:
/ = subject heading
$ = truncation
? = optional wild card character - stands for zero or one character
ti,ab,id = terms in title, abstract or key concept fields
mp = terms in title, abstract, subject heading, key concept or original title fields
adj3 = terms within three words of each other (any order)
adj = terms next to each other (in order specified)

CINAHL Plus
via Ebsco https://www.ebsco.com
Date range: Inception to 20th March 2023
Date searched: 21st March 2023
Records retrieved: 2584

S1 (MH "Orthopedics") 13,691
S2 (MH "Orthopedic Technologists") 97
S3 (MH "Orthopedic Nursing") 2,456
S4 (MH "Orthopedic Care")1,184
S5 TI orthop#edi* OR AB orthop#edi* 42,444
S6 TI ((hand or shoulder or elbow or wrist) N1 surgeon*) OR AB ((hand or shoulder or elbow or wrist) N1 surgeon*) 1,960
S7 TI ((spine or spinal or hip or knee or ankle or foot) N1 surgeon*) OR AB ((spine or spinal or hip or knee or ankle or foot) N1 surgeon*) 2,433
S8 S1 OR S2 OR S3 OR S4 OR S5 OR S6 OR S7 55,754
S9 (MH "Physical Therapists") 14,472
S10 (MH "Podiatrists") 2,669
S11 TI ((physical N1 therapist*) or physiotherapist*) OR AB ((physical N1 therapist*) or physiotherapist*) 19,125
S12 TI hand N1 therapist* OR AB hand N1 therapist*472
S13 TI podiatrist* OR AB podiatrist* 2,633
S14 S9 OR S10 OR S11 OR S12 OR S13 32,783
S15 TI (surgery or surgeries or surgical* or operation* or operate* or operative*) OR AB (surgery or surgeries or surgical* or operation* or operate* or operative*) OR SU (surgery or surgeries or surgical* or operation* or operate* or operative*) 842,605
S16 S14 AND S15 2,481
S17 S8 OR S16 57,765
S18 (MH "Decision Making")59,016
S19 (MH "Decision Making, Clinical+") 37,532
S20 (MH "Decision Making, Shared") 3,201
S21 TI (decision* N2 (making or make* or made or maker*)) OR AB (decision* N2 (making or make* or made or maker*)) 103,664
S22 TI (decision* N3 (clinical or evidence or knowledge or practice* or procedure* or surgeon* or surgery or surgeries or surgical* or operation* or operate* or operative* or treatment* or

technique* or manag* or care)) OR AB (decision* N3 (clinical or evidence or knowledge or practice* or procedure* or surgeon* or surgery or surgeries or surgical* or operation* or operate* or operative* or treatment* or technique* or manag* or care)) 60,879
S23 TI (decision* N2 (share* or sharing)) OR AB (decision* N2 (share* or sharing)) 8,591
S24 S18 OR S19 OR S20 OR S21 OR S22 OR S23 191,794
S25 S17 AND S24 2,246
S26 (MH "Practice Patterns") 13,514
S27 (MH "Professional Practice") OR (MH "Professional Autonomy") 24,299
S28 TI ((variation* or vary* or varies or varied* or variability*) N3 (practice* or procedure* or surgeon* or surgery or surgeries or surgical* or operation* or operate* or operative* or treatment* or technique* or manag* or care)) OR AB ((variation* or vary* or varies or varied* or variability*) N3 (practice* or procedure* or surgeon* or surgery or surgeries or surgical* or operation* or operate* or operative* or treatment* or technique* or manag* or care)) 23,091
S29 TI ((variation* or vary* or varies or varied* or variability*) N2 (local* or regional* or national* or international* or country or countries or geographic*)) OR AB ((variation* or vary* or varies or varied* or variability*) N2 (local* or regional* or national* or international* or country or countries or geographic*)) 10,966
S30 TI (practice* N2 (pattern* or trend*)) OR AB (practice* N2 (pattern* or trend*)) 6,353
S31 S26 OR S27 OR S28 OR S29 OR S30 73,907
S32 S17 AND S31 1,371
S33 (MH "Professional Practice, Evidence-Based+") 85,471
S34 TI (evidence-based N1 (practice* or medicine or nursing or treatment* or surgery or care)) OR AB (evidence-based N1 (practice* or medicine or nursing or treatment* or surgery or care)) OR TI (evidence-based N2 (paediatric* or pediatric* or "child health")) OR AB (evidence-based N2 (paediatric* or pediatric* or "child health")) 34,967
S35 TI (evidencebased N1 (practice* or medicine or nursing or treatment* or surgery or care)) OR AB (evidencebased N1 (practice* or medicine or nursing or treatment* or surgery or care)) OR TI (evidencebased N2 (paediatric* or pediatric* or "child health")) OR AB (evidencebased N2 (paediatric* or pediatric* or "child health")) 196
S36 S33 OR S34 OR S35 103,315
S37 S17 AND S36 1,117
S38 S25 OR S32 OR S37 4,315
S39 TI decision* N3 orthop#edi* OR AB decision* N3 orthop#edi* 150
S40 TI ((variation* or vary* or varies or varied* or variability*) N3 orthop#edi*) OR AB ((variation* or vary* or varies or varied* or variability*) N3 orthop#edi*) 168
S41 TI evidence-based N1 orthop#edi* OR AB evidence-based N1 orthop#edi* OR TI evidencebased N1 orthop#edi* OR AB evidencebased N1 orthop#edi* 72
S42 S39 OR S40 OR S41 388
S43 S38 OR S42 4,416
S44 S38 OR S42 Limiters - Published Date: 20140101-20230431; English 2,591
S45 MH animals+ 105,628
S46 MH (animal studies) 152,109
S47 TI (animal model*) 3,774
S48 S45 OR S46 OR S47 248,854
S49 MH (human) 2,689,326
S50 S48 NOT S49 214,801
S51 S44 NOT S50 2,584

Key
MH = subject heading

MH + = exploded subject heading
* = truncation
# = optional wild card character - stands for zero or one character
TI = title search
AB = abstract search
SU = subject heading search
N3 = terms within three words of each other (any order)

ASSIA
via ProQuest https://www.proquest.com
Date range: Inception to 20th March 2023
Date searched: 21st March 2023
Records retrieved: 189

The MEDLINE strategy was modified to fit the ProQuest ASSIA search interface. 7 search lines were used and results from each line were downloaded into EndNote for duplicate removal.

1. (TI,AB,SU,IF(orthop?edi*) OR (TI,AB,SU,IF((hand OR shoulder OR elbow OR wrist) NEAR/0 surgeon*) OR TI,AB,SU,IF((spine OR spinal OR hip OR knee OR ankle OR foot) NEAR/0 surgeon*)) OR (TI,AB,SU,IF((physical NEAR/0 therapist*) OR physiotherapist*) OR TI,AB,SU,IF(hand NEAR/0 therapist*) OR TI,AB,SU,IF(podiatrist*))) AND (TI,AB,SU,IF(decision* NEAR/2 (making OR make? OR made OR maker?)) OR TI,AB,SU,IF(decision* NEAR/3 (clinical OR evidence OR knowledge OR practice* OR procedure* OR surgeon* OR surgery OR surgeries OR surgical* OR operation* OR operate* OR operative* OR treatment* OR technique* OR manag* OR care)) OR TI,AB,SU,IF(decision* NEAR/2 (share* OR sharing))) limits: 2014-01-01 to 2023-03-21, English language
89 hits

2. (TI,AB,SU,IF(orthop?edi*) OR (TI,AB,SU,IF((hand OR shoulder OR elbow OR wrist) NEAR/0 surgeon*) OR TI,AB,SU,IF((spine OR spinal OR hip OR knee OR ankle OR foot) NEAR/0 surgeon*)) OR (TI,AB,SU,IF((physical NEAR/0 therapist*) OR physiotherapist*) OR TI,AB,SU,IF(hand NEAR/0 therapist*) OR TI,AB,SU,IF(podiatrist*))) AND TI,AB,SU,IF(practice* NEAR/2 (pattern* OR trend*)) limits: 2014-01-01 to 2023-03-21, English language
6 hits

3. (TI,AB,SU,IF(orthop?edi*) OR (TI,AB,SU,IF((hand OR shoulder OR elbow OR wrist) NEAR/0 surgeon*) OR TI,AB,SU,IF((spine OR spinal OR hip OR knee OR ankle OR foot) NEAR/0 surgeon*)) OR (TI,AB,SU,IF((physical NEAR/0 therapist*) OR physiotherapist*) OR TI,AB,SU,IF(hand NEAR/0 therapist*) OR TI,AB,SU,IF(podiatrist*))) AND TI,AB,SU,IF((variation* OR vary* OR varies OR varied* OR variability*) NEAR/3 (practice* OR procedure* OR surgeon* OR surgery OR surgeries OR surgical* OR operation* OR operate* OR operative* OR treatment* OR technique* OR manag* OR care)) limits: 2014-01-01 to 2023-03-21 , English language
19 hits

4. (TI,AB,SU,IF(orthop?edi*) OR (TI,AB,SU,IF((hand OR shoulder OR elbow OR wrist) NEAR/0 surgeon*) OR TI,AB,SU,IF((spine OR spinal OR hip OR knee OR ankle OR foot) NEAR/0 surgeon*)) OR (TI,AB,SU,IF((physical NEAR/0 therapist*) OR physiotherapist*) OR TI,AB,SU,IF(hand NEAR/0 therapist*) OR TI,AB,SU,IF(podiatrist*))) AND TI,AB,SU,IF((variation* OR vary* OR varies OR varied* OR variability*) NEAR/3 (local* OR regional* OR national* OR international* OR country OR countries OR geographic*)) limits: 2014-01-01 to 2023-03-21, English language
5 hits

5. (TI,AB,SU,IF(orthop?edi*) OR (TI,AB,SU,IF((hand OR shoulder OR elbow OR wrist) NEAR/0 surgeon*) OR TI,AB,SU,IF((spine OR spinal OR hip OR knee OR ankle OR foot) NEAR/0 surgeon*)) OR (TI,AB,SU,IF((physical NEAR/0 therapist*) OR physiotherapist*) OR TI,AB,SU,IF(hand NEAR/0 therapist*) OR TI,AB,SU,IF(podiatrist*))) AND TI,AB,SU,IF((evidence-based OR evidencebased) NEAR/0 (practice* OR medicine OR nursing OR treatment* OR surgery OR care)) limits: 2014-01-01 to 2023-03-21, English language
67 hits

6. TI,AB,SU,IF(decision* NEAR/3 orthop?edi*) OR TI,AB,SU,IF((variation* OR vary* OR varies OR varied* OR variability*) NEAR/3 orthop?edi*) OR TI,AB,SU,IF((evidence-based OR evidencebased) NEAR/0 orthop?edi*) limits: 2014-01-01 to 2023-03-21, English language
3 hits

7. (TI,AB,SU,IF(orthop?edi*) OR (TI,AB,SU,IF((hand OR shoulder OR elbow OR wrist) NEAR/0 surgeon*) OR TI,AB,SU,IF((spine OR spinal OR hip OR knee OR ankle OR foot) NEAR/0 surgeon*)) OR (TI,AB,SU,IF((physical NEAR/0 therapist*) OR physiotherapist*) OR TI,AB,SU,IF(hand NEAR/0 therapist*) OR TI,AB,SU,IF(podiatrist*))) AND TI,AB,SU,IF((evidence-based OR evidencebased) NEAR/2 (paediatric* OR pediatric* OR "child health")) limits: 2014-01-01 to 2023-03-21, English language
0 hits

Key:
* = truncation
? = optional wild card character - stands for zero or one character
TI,AB,SU,IF = search of title, abstract, subject heading or keyword fields
NEAR/3 = terms within three words of each other (any order)
NEAR/0 = terms next to each other (any order)

Science Citation Index
Date range: 1900-20th March 2023
Social Science Citation Index
Date range: 1956-20th March 2023
via Web of Science, Clarivate https://clarivate.com
Date searched: 21st March 2023
Records retrieved: 3088

Settings: Exact search switched on

1: TS=(orthop$edi*) Editions: WOS.SSCI,WOS.SCI Results: 93079
2: TS=((hand or shoulder or elbow or wrist) NEAR/0 surgeon*) Editions: WOS.SSCI,WOS.SCI Results: 3956
3: TS=((spine or spinal or hip or knee or ankle or foot) NEAR/0 surgeon*) Editions: WOS.SSCI,WOS.SCI Results: 5948
4: #1 OR #2 OR #3 Editions: WOS.SSCI,WOS.SCI Results: 101340
5: TS=((physical NEAR/0 therapist*) or physiotherapist*) Editions: WOS.SSCI,WOS.SCI Results: 14277
6: TS=(hand NEAR/0 therapist*) Editions: WOS.SSCI,WOS.SCI Results: 247
7: TS=(podiatrist*) Editions: WOS.SSCI,WOS.SCI Results: 695
8: #5 OR #6 OR #7 Editions: WOS.SSCI,WOS.SCI Results: 15124

9: TS=(surgery or surgeries or surgical* or operation* or operate* or operative*) Editions: WOS.SSCI,WOS.SCI Results: 3271941

10: #8 AND #9 Editions: WOS.SSCI,WOS.SCI Results: 1994
11: #10 OR #4 Editions: WOS.SSCI,WOS.SCI Results: 102865
12: TS=(decision* NEAR/2 (making or make$ or made or maker$)) Editions: WOS.SSCI,WOS.SCI Results: 488938
13: TS=(decision* NEAR/3 (clinical or evidence or knowledge or practice* or procedure* or surgeon* or surgery or surgeries or surgical* or operation* or operate* or operative* or treatment* or technique* or manag* or care)) Editions: WOS.SSCI,WOS.SCI Results: 174395
14: TS=(decision* NEAR/2 (share* or sharing)) Editions: WOS.SSCI,WOS.SCI Results: 17019
15: #14 OR #13 OR #12 Editions: WOS.SSCI,WOS.SCI Results: 567485
16: #15 AND #11 Editions: WOS.SSCI,WOS.SCI Results: 2784
17: TS=((variation* or vary* or varies or varied* or variability*) NEAR/3 (practice* or procedure* or surgeon* or surgery or surgeries or surgical* or operation* or operate* or operative* or treatment* or technique* or manag* or care)) Editions: WOS.SSCI,WOS.SCI Results: 90289
18: TS=((variation* or vary* or varies or varied* or variability*) NEAR/2 (local* or regional* or national* or international* or country or countries or geographic*)) Editions: WOS.SSCI,WOS.SCI Results: 94402
19: TS=(practice* NEAR/2 (pattern* or trend*)) Editions: WOS.SSCI,WOS.SCI Results: 18301
20: #17 OR #18 OR #19 Editions: WOS.SSCI,WOS.SCI Results: 197396
21: #20 AND #11 Editions: WOS.SSCI,WOS.SCI Results: 1690
22: TS=((evidence-based or evidencebased) NEAR/0 (practice* or medicine or nursing or treatment* or surgery or care)) Editions: WOS.SSCI,WOS.SCI Results: 44242
23: TS=((evidence-based or evidencebased) NEAR/2 (paediatric* or pediatric* or "child health")) Editions: WOS.SSCI,WOS.SCI Results: 605
24: #22 OR #23 Editions: WOS.SSCI,WOS.SCI Results: 44601
25: #24 AND #11 Editions: WOS.SSCI,WOS.SCI Results: 569
26: TS=(decision* NEAR/3 orthop$edi*) Editions: WOS.SSCI,WOS.SCI Results: 202
27: TS=((variation* or vary* or varies or varied* or variability*) NEAR/3 orthop$edi*) Editions: WOS.SSCI,WOS.SCI Results: 244
28: TS=((evidence-based or evidencebased) NEAR/0 orthop$edi*) Editions: WOS.SSCI,WOS.SCI Results: 76
29: #26 OR #27 OR #28 Editions: WOS.SSCI,WOS.SCI Results: 518
30: #29 OR #25 OR #21 OR #16 Editions: WOS.SSCI,WOS.SCI Results: 4870
31: #29 OR #25 OR #21 OR #16 and 2023 or 2022 or 2021 or 2020 or 2019 or 2018 or 2017 or 2016 or 2015 or 2014 (Publication Years) Editions: WOS.SSCI,WOS.SCI Results: 3153
32: #29 OR #25 OR #21 OR #16 and 2023 or 2022 or 2021 or 2020 or 2019 or 2018 or 2017 or 2016 or 2015 or 2014 (Publication Years) and English (Languages) Editions: WOS.SSCI,WOS.SCI Results: 3088

Key:
* = truncation
$ = optional wild card character - stands for zero or one character
TS = topic tag; searches in title, abstract, author keywords and keywords plus fields
NEAR/3 = terms within three words of each other (any order)
NEAR/0 = terms next to each other (any order)

Cochrane Central Register of Controlled Trials (CENTRAL)
via Wiley http://onlinelibrary.wiley.com/
Issue 3 of 12, March 2023
Date searched: 21st March 2023
Records retrieved: 619

#1 MeSH descriptor: [Orthopedics] this term only 492
#2 MeSH descriptor: [Orthopedic Surgeons] this term only 32
#3 orthop?edi*:ti,ab,kw 14256
#4 ((hand or shoulder or elbow or wrist) next surgeon*):ti,ab,kw 503
#5 ((spine or spinal or hip or knee or ankle or foot) next surgeon*):ti,ab,kw 260
#6 #1 or #2 or #3 or #4 or #5 14859
#7 MeSH descriptor: [Physical Therapists] this term only 205
#8 MeSH descriptor: [Podiatry] this term only 48
#9 (physical next therapist* or physiotherapist*):ti,ab,kw 8571
#10 (hand next therapist*):ti,ab,kw 93
#11 podiatrist*:ti,ab,kw 159
#12 #7 or #8 or #9 or #10 or #11 8816
#13 (surgery or surgeries or surgical* or operation* or operate* or operative*) 356041
#14 #12 and #13 1883
#15 #6 or #14 16450
#16 MeSH descriptor: [Decision Making] this term only 3008
#17 MeSH descriptor: [Clinical Decision-Making] explode all trees 529
#18 MeSH descriptor: [Decision Making, Shared] this term only 136
#19 (decision* near/2 (making or make? or made or maker?)):ti,ab,kw 20469
#20 (decision* near/3 (clinical or evidence or knowledge or practice* or procedure* or surgeon* or surgery or surgeries or surgical* or operation* or operate* or operative* or treatment* or technique* or manag* or care)):ti,ab,kw13924
#21 (decision* near/2 (share* or sharing)):ti,ab,kw 2068
#22 #16 or #17 or #18 or #19 or #20 or #21 26545
#23 #15 and #22 428
#24 MeSH descriptor: [Practice Patterns, Physicians'] this term only 1765
#25 MeSH descriptor: [Practice Patterns, Nurses'] this term only 185
#26 MeSH descriptor: [Professional Practice] this term only 155
#27 MeSH descriptor: [Professional Autonomy] this term only 37
#28 ((variation* or vary* or varies or varied* or variability*) near/3 (practice* or procedure* or surgeon* or surgery or surgeries or surgical* or operation* or operate* or operative* or treatment* or technique* or manag* or care)):ti,ab,kw 4987
#29 ((variation* or vary* or varies or varied* or variability*) near/2 (local* or regional* or national* or international* or country or countries or geographic*)):ti,ab,kw 848
#30 (practice* near/2 (pattern* or trend*)):ti,ab,kw 2609
#31 #24 or #25 or #26 or #27 or #28 or #29 or #30 8373
#32 #15 and #31 194
#33 MeSH descriptor: [Evidence-Based Practice] this term only 456
#34 MeSH descriptor: [Evidence-Based Medicine] this term only 2966
#35 MeSH descriptor: [Evidence-Based Nursing] this term only 75
#36 ((evidence-based or evidencebased) next (practice* or medicine or nursing or treatment* or surgery or care)):ti,ab,kw 10732
#37 ((evidence-based or evidencebased) near/2 (paediatric* or pediatric* or "child health")):ti,ab,kw 20
#38 #33 or #34 or #35 or #36 or #37 10741

#39 #15 and #38 156
#40 #23 or #32 or #39 704
#41 (decision* near/3 orthop?edi*):ti,ab,kw 16
#42 ((variation* or vary* or varies or varied* or variability*) near/3 orthop?edi*):ti,ab,kw 13
#43 ((evidence-based or evidencebased) next orthop?edi*):ti,ab,kw 6
#44 #41 or #42 or #43 34
#45 #40 or #44 713
#46 #40 or #44 with Cochrane Library publication date Between Jan 2014 and Apr 2023, in Trials 619

Key:
MeSH descriptor = subject heading
* = truncation
? = optional wild card character - stands for zero or one character
ti,ab,kw = terms in title, abstract or keyword fields
near/3 = terms within three words of each other (any order)
next = terms are next to each other

Cochrane Database of Systematic Reviews (CDSR)
via Wiley http://onlinelibrary.wiley.com/
Issue 3 of 12, March 2023
Date searched: 21st March 2023
Records retrieved: 10

#1 MeSH descriptor: [Orthopedics] this term only 492
#2 MeSH descriptor: [Orthopedic Surgeons] this term only 32
#3 orthop?edi*:ti,ab,kw 14256
#4 ((hand or shoulder or elbow or wrist) next surgeon*):ti,ab,kw 503
#5 ((spine or spinal or hip or knee or ankle or foot) next surgeon*):ti,ab,kw 260
#6 #1 or #2 or #3 or #4 or #5 14859
#7 MeSH descriptor: [Physical Therapists] this term only 205
#8 MeSH descriptor: [Podiatry] this term only 48
#9 (physical next therapist* or physiotherapist*):ti,ab,kw 8571
#10 (hand next therapist*):ti,ab,kw 93
#11 podiatrist*:ti,ab,kw 159
#12 #7 or #8 or #9 or #10 or #11 8816
#13 (surgery or surgeries or surgical* or operation* or operate* or operative*) 356041
#14 #12 and #13 1883
#15 #6 or #14 16450
#16 MeSH descriptor: [Decision Making] this term only 3008
#17 MeSH descriptor: [Clinical Decision-Making] explode all trees 529
#18 MeSH descriptor: [Decision Making, Shared] this term only 136
#19 (decision* near/2 (making or make? or made or maker?)):ti,ab,kw 20469
#20 (decision* near/3 (clinical or evidence or knowledge or practice* or procedure* or surgeon* or surgery or surgeries or surgical* or operation* or operate* or operative* or treatment* or technique* or manag* or care)):ti,ab,kw13924
#21 (decision* near/2 (share* or sharing)):ti,ab,kw 2068
#22 #16 or #17 or #18 or #19 or #20 or #21 26545
#23 #15 and #22 428
#24 MeSH descriptor: [Practice Patterns, Physicians'] this term only 1765
#25 MeSH descriptor: [Practice Patterns, Nurses'] this term only 185

#26 MeSH descriptor: [Professional Practice] this term only 155
#27 MeSH descriptor: [Professional Autonomy] this term only 37
#28 ((variation* or vary* or varies or varied* or variability*) near/3 (practice* or procedure* or surgeon* or surgery or surgeries or surgical* or operation* or operate* or operative* or treatment* or technique* or manag* or care)):ti,ab,kw 4987
#29 ((variation* or vary* or varies or varied* or variability*) near/2 (local* or regional* or national* or international* or country or countries or geographic*)):ti,ab,kw 848
#30 (practice* near/2 (pattern* or trend*)):ti,ab,kw 2609
#31 #24 or #25 or #26 or #27 or #28 or #29 or #30 8373
#32 #15 and #31 194
#33 MeSH descriptor: [Evidence-Based Practice] this term only 456
#34 MeSH descriptor: [Evidence-Based Medicine] this term only 2966
#35 MeSH descriptor: [Evidence-Based Nursing] this term only 75
#36 ((evidence-based or evidencebased) next (practice* or medicine or nursing or treatment* or surgery or care)):ti,ab,kw 10732
#37 ((evidence-based or evidencebased) near/2 (paediatric* or pediatric* or "child health")):ti,ab,kw 20
#38 #33 or #34 or #35 or #36 or #37 10741
#39 #15 and #38 156
#40 #23 or #32 or #39 704
#41 (decision* near/3 orthop?edi*):ti,ab,kw 16
#42 ((variation* or vary* or varies or varied* or variability*) near/3 orthop?edi*):ti,ab,kw 13
#43 ((evidence-based or evidencebased) next orthop?edi*):ti,ab,kw 6
#44 #41 or #42 or #43 34
#45 #40 or #44 713
#46 #40 or #44 with Cochrane Library publication date Between Jan 2014 and Apr 2023, in Cochrane Reviews, Cochrane Protocols 10

Key:
MeSH descriptor = subject heading
* = truncation
? = optional wild card character - stands for zero or one character
ti,ab,kw = terms in title, abstract or keyword fields
near/3 = terms within three words of each other (any order)
next = terms are next to each other
