# Supplementary material for: Can we ever have evidence-based decision making in orthopaedics? A qualitative evidence synthesis and conceptual framework
Source: BMC Med Inform Decis Mak. 2025 Jul 1;25:216. doi: 10.1186/s12911-025-03032-5 (PMC12211141; doi:10.1186/s12911-025-03032-5)
Supplement: Supplementary file 4 — Supplementary Material 4: Quality appraisal for included studies using CASP tool [file 12911_2025_3032_MOESM4_ESM.pdf]

Additional file 4 Quality appraisal for included studies using CASP [20]

Author	Q1	Q2	Q3	Q4	Q5	Q6	Q7	Q8	Q9	CASP Appraisal
Adogwa et al (2021)	Yes	Yes	Yes	Yes	Yes	No	Yes	Yes	No	Valuable
Baker et al (2019)	Yes	Yes	No	Yes	Yes	No	No	Unclear	Yes	Unclear
Barton et al (2021)	Yes	Yes	Yes	Yes	Yes	No	Yes	Yes	Yes	Valuable
Brown et al (2018)	Yes	Yes	Yes	Yes	No	Yes	Yes	Yes	Yes	Valuable
Bunzil et al (2017)	Yes	Yes	No	Yes	Yes	Yes	Yes	Yes	Unclear	Valuable
Bunzil et al (2021)	Yes	Yes	Yes	Yes	Yes	No	Yes	Yes	Unclear	Valuable
Coole et al (2021)	Yes	Yes	Yes	Yes	Yes	No	Yes	Yes	Yes	Valuable
Dismore et al (2021)	Yes	Yes	Yes	Yes	Yes	No	Yes	Yes	Yes	Valuable
Frankel et al (2016)	Yes	Yes	Yes	Yes	Yes	No	Yes	Unclear	Yes	Valuable
Grove et al (201 8)	Yes	Yes	Yes	Yes	Yes	No	Yes	Yes	Yes	Valuable
Grove et al (2020)	Yes	Yes	Yes	Yes	Yes	No	Yes	Yes	Yes	Valuable
Grove et al (2021)	Yes	Yes	Yes	Yes	Unclear	No	Yes	Yes	Yes	Valuable
Haider et al (2020)	Yes	Yes	Yes	Yes	Yes	No	No	Yes	Yes	Unclear
Hsu et al (2017)	Yes	Yes	Yes	Yes	Yes	No	No	Yes	Yes	Valuable
Jefferson et al (2017)	Yes	Yes	Yes	Yes	Yes	Yes	Yes	Yes	Yes	Valuable
Madsen et al (2021)	Yes	Yes	Yes	Yes	No	Yes	Yes	Yes	Yes	Valuable
Moore et al (2017)	Yes	Yes	Yes	Yes	Yes	No	Yes	Yes	Yes	Valuable
Phelps et al (2019)	Yes	Yes	Yes	Yes	Yes	Yes	Yes	Yes	Yes	Valuable
Rath et al (2017)	Yes	Yes	Yes	Yes	Yes	No	Yes	Yes	Yes	Valuable
Rehman et al (2019)	Yes	Yes	Yes	Yes	Yes	No	Yes	Yes	Yes	Valuable

Robba et al (2019)	Yes	Yes	Yes	Yes	Yes	No	Yes	Yes	Yes	Valuable
Scantlebury et al (2022)	Yes	Yes	Yes	Yes	Yes	No	Yes	Yes	Yes	Valuable
Schmidtke et al (2022)	Yes	Yes	Yes	Yes	Yes	No	Yes	Yes	Yes	Valuable
Shaw et al (2022)	Yes	Yes	Yes	Yes	Yes	No	Yes	Yes	Yes	Valuable
Sutton et al (2021)	Yes	Yes	Yes	Yes	Yes	Yes	Yes	Yes	Yes	Valuable
