# Supplementary material for: Can we ever have evidence-based decision making in orthopaedics? A qualitative evidence synthesis and conceptual framework
Source: BMC Med Inform Decis Mak. 2025 Jul 1;25:216. doi: 10.1186/s12911-025-03032-5 (PMC12211141; doi:10.1186/s12911-025-03032-5)
Supplement: Supplementary file 5 — Supplementary Material 5: Exemplar quotes for thematic synthesis [file 12911_2025_3032_MOESM5_ESM.pdf]

Additional file 5: Exemplar quotes for thematic synthesis

Source of knowledge/evidence	Exemplar quote(s)
**Formal codified knowledge**	*"There are people who don't follow policy…evidence-based guidance you know. I think it's an orthopaedic tradition, it's just more characteristic of us [orthopaedic surgeons]." Grove et al., 2022*<br>*""Radiation oncology is extremely…guideline-oriented…all [specialties involved in oncology care] are, but it's almost hyper." "If any of those 2 factors are prompted…[Spinal Instability Neoplastic Score] above a 7, and/or any evidence of epidural tumor causing [neurologic] compression, then immediately we're thinking that [a] surgeon needs to fully evaluate the patients." Barton et al., 2021*
**Informal experiential, implicit knowledge**	*My bias would be that, if I just intuitively if you said, 'What are you doing for this patient?' I'd just go, 'two-stage' because that's what I've always done. That's what I've been taught. That's what I've read about. And that's what appears to be slightly better. But I realise that scientifically there's no big comparative trial.*<br>*Well, I suppose the truth is, we do not know which technique, one or two-stage, is better. We know the two-stage probably has a slightly better chance of eradication of infection than one-stage by about 10%, so that is the reason to do it. (Moore et al., 2017)*<br>*"I trust [the research] but I want my data no doubt about it because I think I am better… I know lots of faults in techniques or little things that really can comprise outcome. So everyone has a different hand and surgery is very touchy practice…. So I believe what happens around but at the same time I want mine as well because I know what I do differently or I am more careful about." (Bunzli et al., 2017)*
**Individual patient and surgeon characteristics**	*"There maybe two factors… the family may be of the perception that it is a lost game and there is no point wasting money and let the person be at home. Another may be that our health system probably chooses the younger patient who has a better chance of recovery than in comparison to the older person". (Rath et al., 2017)*<br>*"I received a lot of pressure from my family saying, "don't go to that surgery, you are going to be contaminated by the virus," and another pressure from the father's patient, saying to me, "No, doctor. I need you to be the surgeon." (Bunzli et al., 2021)*<br>*"But you get people who don't have a massive amount of pain, are functionally pretty good, but once people are on a pathway it seems nobody puts up the stop sign. And I do wonder with a small group of patients if they should wait a little longer" (Coole et al., 2021)*
**Managerial Knowledge**	*"Probably, the hospital gets paid more for doing a two stage, right, which is always at the back of the surgeon's mind […] the quality of patient care you can give, depends on your Trust not being in the red. So, you don't want to do operations that lose your Trust money but your first priority is to get the best for the patient. Plus, when you're in indecision, the fact that the two-stage operation doesn't cost – that doesn't, you know, it brings in more resources to the Trust, means you feel more relaxed about that option." (Moore et al., 2017)*
**Organisational Knowledge**	*"We didn't until recently have the appropriate infrastructure in [our hospital] with regard to the microbiology department in order to be able to identify organisms accurately prior to surgery […] more often than not the*

	aspirations would either come back negative or the laboratory would have some excuse as to why the sample hasn't been tested properly […] Now over the last 2 years the microbiology department has changed and there's a new set of individuals there who are more proactive and much more amenable to discussing matters with the clinician." (Moore et al., 2017)
**Socialisation and association with other colleagues**	*"I think there's a big difference between having a radiation oncologist that we know, that we've worked with because you can kind of finish each other sentences a little bit, and so when they present to you this patient is surgical, they know you. You know them, and if they're saying it, it becomes a different situation than if you're told from somebody you don't know, a radiation oncologist.'" (Barton et al., 2021)*<br>*"I think you are heavily influenced by your peers. But you are working in the confines of a hospital, so the more you work together the less novel your behaviour becomes…it's more standardised." (Grove et al., 2022)*
**Cultural normative and political influence norms**	*"I think they have a very low threshold for operating because they feel more exposed. If you do nothing, you're obviously more exposed than if you do something, even if you do something wrong." (Scantlebury et al., 2022)*
**Training and education**	*"I think in most cases it's a product of who you trained with, what you've seen and actually I'm sure people have said it but one of the most powerful things is probably the "Journal of Anecdotal Medicine" and burnt fingers but I think you can have one case that you get your fingers burnt with doing one thing or another and it's very hard to look past, oh I did this and we had a really bad experience. As a clinician it's sometimes very hard to unsee that."* (Scantlebury et al., 2022).
