# Supplementary material for: Can we ever have evidence-based decision making in orthopaedics? A qualitative evidence synthesis and conceptual framework
Source: BMC Med Inform Decis Mak. 2025 Jul 1;25:216. doi: 10.1186/s12911-025-03032-5 (PMC12211141; doi:10.1186/s12911-025-03032-5)
Supplement: Supplementary file 6 — Supplementary Material 6: Conceptual Framework of the sources of evidence and drivers of variation in orthopaedic surgical work (Grove et al., 2016 versus. Conceptual Framework of Evidence-Based Orthopaedics: The Linchpin Model [file 12911_2025_3032_MOESM6_ESM.pdf]

Appendix 6. Conceptual Framework of the sources of evidence and drivers of variation in orthopaedic surgical work (Grove et al., 2016 versus. Conceptual Framework of Evidence-Based Orthopaedics: The Linchpin Model

**Driver of surgical decision making**	**Conceptual Framework of Evidence-Based Orthopaedics: The Linchpin Model**	**Conceptual Framework of the sources of evidence and drivers of variation in orthopaedic surgical work**
Surgeon identity	Surgeon identity can explain different responses to standardisation and the extent that evidence is used to guide decision-making.<br><br>Our re-conceptualisation poses that surgeon identity is an explanatory linchpin which mediates the other drivers of decision-making.	Not present
Organisational capacity	The way we do things around here. The culture of evidence-based practice within an organisation and organisation type influence the use of evidence in decision-making. *Where* patients receive treatment drives decision-making.<br><br>Our revised framework describes organisational capacity as an explanatory linchpin for understanding how decisions are made, which includes, but expands upon resources.	Organisational knowledge was conceptualised as a distinct driver of decision-making and focussed on resource availability.
Formal codified knowledge	Macro level clinical guidelines and scientific literature.<br>Our re-conceptualisation demonstrates how variation in the perception and application of formal codified knowledge may be explained by surgeon identity and organisational capacity.	The original framework highlighted the extent that included studies reported that formal codified knowledge influenced surgical decisions.
Informal experiential knowledge	Our revised framework expands on the notion that the tacit knowledge surgeon's build up over time, but which is difficult to describe is fundamental to decision-making. We highlight that relying on experience and the skill of surgeons and the need to tailor decisions to individual patients legitimises resistance to	"Clinical intuition" and surgical experience. A surgeon's gut feeling provides an ability to predict which patients may do well and/or who may benefit from surgical intervention.

	evidence based practice as it questions the applicability of standardised evidence and guidelines (e.g. randomised controlled trials) to surgical work.	
Managerial knowledge	The cost of orthopaedic work – healthcare finance, the cost of orthopaedic provision and how this influences surgical practice.	Defined as resource issues – time, cost and the safety and quality of services. Qualitative evidence was focussed towards the cost of orthopaedic services and financial competition.
Organisational knowledge	This has been reconceptualised and included within managerial knowledge and organisational capacity.	Originally conceptualised as an individual driver of decision-making, organisational knowledge shapes the perspectives of clinicians working in an organisation – "the way we do things around here."
Socialisation and association with colleagues	Orthopaedic surgery is hierarchical in nature. Evidence becomes legitimate surgical knowledge when it is developed and defined by individuals within a surgeon's specialty or sub-specialty.<br><br>Our reconceptualization highlights that evidence produced by respected individuals and/or national societies whilst accepted is insufficient as a lever for individual or organisational level practice change in isolation.	Highlights a strong elitist community of practice within orthopaedics and distrust or dismissal of evidence that is produced from outside the profession.
Individual patient and surgeon factors	The characteristics of patients and surgeons that influence decision-making.<br>The process of shared-decision making between patients and surgeons was included within our re-conceptualisation, which did not feature within the original framework.	Emphasised age-related factors and patients experiences of pain in surgical decision making. Comparatively little qualitative evidence described the influence of surgeon demographics on decision-making.
Culture, norms and political influence of the sector	The wider orthopaedic profession and how national standards, regulation and government policies can influence evidence adoption.<br><br>Our qualitative data, also highlights the role of cultural expectations to conform to professional expectations (i.e.	Focusses on how professional societies (e.g. British Orthopaedic Association UK) helps to retain autonomy and protect orthopaedic practices from external intervention – e.g. clinical guidelines produced outside the orthopaedic sector.

	operate) particularly in areas where there is limited formal codified knowledge.<br><br>Both frameworks highlight a perceived threat of medico-legal challenge and peer judgement as a driver for surgical intervention.	
Training and formal education	Surgical training and education are considered foundations of surgical work, which provide surgeons with a philosophy of practice closely tied to implicit knowledge.<br><br>We expand previous thinking to link training and education and surgical philosophy/experiential knowledge. Both conceptualisations suggest that whilst fundamental, knowledge obtained through training is flexible and can be replaced.	Outlines the type of training and formal education surgeons obtain.
