# Supplementary material for: Can we ever have evidence-based decision making in orthopaedics? A qualitative evidence synthesis and conceptual framework
Source: BMC Med Inform Decis Mak. 2025 Jul 1;25:216. doi: 10.1186/s12911-025-03032-5 (PMC12211141; doi:10.1186/s12911-025-03032-5)
Supplement: Supplementary file 7 — Supplementary Material 7: Patient related factors [file 12911_2025_3032_MOESM7_ESM.pdf]

Additional file 7: Patient related factors influencing decision-making

Patient related factors influencing decision making as described by the original author	
**Patient demographic characteristics**	• General statements about the influence of: medical history, comorbidities, overall health and life expectancy and 'fitness for surgery' [28, 29, 32, 36, 38, 39, 41]<br>• Mental health and Dementia [41]<br>• Drug use [41]<br>• Frailty and age [36]<br>• Weight [41]<br>• Mobility [27, 29, 32]<br>• Employment (e.g. self-employed particularly motivated for quick recovery; delaying surgery until retirement) [7, 35]<br>• Socioeconomic status [28, 38, 29]<br>• Cultural beliefs [42]<br>• Health literacy [28]<br>• Diet [41]<br>• Social circumstances and/or support; particularly post-operative support. [28, 29, 38]<br>• Quality of life and activities of daily living [28, 29, 38]<br>• Sleep quality [39]
**Bone characteristics**	• Polytrauma [7]<br>• Functional status [38, 39]<br>• Osteoporosis [7, 38]<br>• Arthritis [41]<br>• Condition of bone and surrounding tissue [29, 38]<br>• Neurological deficit [7, 41]<br>• Stability [33] and Spinal stability [7, 27]<br>• Cemented or uncemented primary fixation[29]<br>• Ambulatory function [29, 41]<br>• Tumour characteristics [27]<br>• Characteristics of infected organism [29]<br>• Imagine and/or radiographic severity [25, 33, 38]<br>• Hyperlaxity [33]<br>• Degenerative change within joint/long-term consequences of chronic Distal Radioulnar joint [33]<br>• To avoid developing kyphosis [7]<br>• Pain [7, 25, 27, 29, 32, 33 38, 39, 40, 41, 42]
**Prior treatments**	• Prior treatments that may reduce surgery effectiveness (e.g. radiation) [27]<br>• Prior conservatives treatment for arthritis [29]<br>• Whether conservative management (e.g. pain killers, pain clinic, physiotherapy) have been tried and were ineffective. [29, 33, 39, 40]
